# Epigenomes in Cardiovascular Disease

**DOI:** 10.1161/CIRCRESAHA.118.311597

**Published:** 2018-05-24

**Authors:** Manuel Rosa-Garrido, Douglas J. Chapski, Thomas M. Vondriska

**Affiliations:** From the Departments of Anesthesiology, Medicine, and Physiology, David Geffen School of Medicine, University of California, Los Angeles.

**Keywords:** cardiovascular diseases, chromatin, epigenomics, genetics, transcriptome

## Abstract

If unifying principles could be revealed for how the same genome encodes different eukaryotic cells and for how genetic variability and environmental input are integrated to impact cardiovascular health, grand challenges in basic cell biology and translational medicine may succumb to experimental dissection. A rich body of work in model systems has implicated chromatin-modifying enzymes, DNA methylation, noncoding RNAs, and other transcriptome-shaping factors in adult health and in the development, progression, and mitigation of cardiovascular disease. Meanwhile, deployment of epigenomic tools, powered by next-generation sequencing technologies in cardiovascular models and human populations, has enabled description of epigenomic landscapes underpinning cellular function in the cardiovascular system. This essay aims to unpack the conceptual framework in which epigenomes are studied and to stimulate discussion on how principles of chromatin function may inform investigations of cardiovascular disease and the development of new therapies.

## Abstract 2

Supplemental Digital Content is available in the text.

The tracks of the train point where it is going—they also reveal where it has been. The tracks of the leopard in the snow do only the latter. Which is the case for epigenetic processes in cardiovascular health and disease? Chromatin enables bespoke storage and retrieval of genetic information across the hundreds of cells in a multicellular organism. Chromatin is also perhaps the largest, in terms of physical size and list of component parts, and most functionally diverse molecular structure in existence, enabling the vast diversity of the multicellular world. The goals of this essay are (1) to review recent advancements in the concepts of epigenomics; (2) to summarize the evidence that epigenomic processes participate in normal and diseased cardiovascular physiology; and (3) to stimulate discussion about the prospects for novel basic science and translational investigations of epigenomes in the cardiovascular system.

In development, the setting in which epigenesis has been most extensively examined, the task of epigenetic processes is to guide the unidirectional differentiation of cells. In adulthood, epigenetic mechanisms provide stability, maintaining the blueprints laid down in development and resisting environmental stresses and stochastic intracellular changes. In cardiovascular disease, an area in which epigenetic processes are increasingly appreciated to operate and in which insights into human health are the most actionable, the task, teleologically, of chromatin is not all that clear. Are the epigenetic processes at work in the cell during disease always combating the insult, attempting to restore and preserve healthy adult phenotypes? Have epigenetic processes been hijacked by evolution to contribute to cell survival decisions? What are the operational principles of epigenetics and how are they distinguished from gene regulation? For this essay, we define an epigenome as a genome plus everything binding to and modifying it (Glossary in the Online Data Supplement).

Evidence has recently accumulated that epigenomic processes play a central role in cardiovascular disease, including (1) genetic and pharmacological gain- and loss-of-function studies in the cardiovascular system targeting individual histone-modifying enzymes, chromatin remodelers, chromatin structural components, and regulatory RNAs can induce or prevent pathology; (2) descriptive epigenomic investigations from humans and animal models demonstrate that widespread reprogramming of histone modifications, DNA methylation, and protein binding occurs during the development of disease; (3) data have arisen that indicate epigenomic processes are interconnected with other cellular events (such as metabolism, differentiation, and cell death) that are deranged in disease and that modulation of these epigenetic processes may hold potential for therapeutic intervention. Let us now examine recent advancements in our understanding of how chromatin controls gene expression and consider the implications for cardiovascular disease.

## Structural Components of Chromatin and Their Regulation in the Cardiovascular System

The epigenome has 2 fundamental components: the structural features of chromatin and the enzymes, RNAs, small molecules, and processes that modify these features. For example, a nucleosome is the chromatin structural unit, but an ATP-dependent chromatin remodeler is an enzyme complex that modifies this structural unit. Here is how you make a nucleosome^1^: bind histone H3 to histone H4 and combine 2 of these dimers to form a tetramer; then combine this tetramer with histone H2A and histone H2B, twice, and embrace this octamer in turn with ≈145 to 147 bp of DNA. Crystal structures^2^ of the nucleosome in 1997 revealed this octameric protein complex to be assembled through histone fold dimers of H2A with H2B and H3 with H4. The intervening years have seen the discovery of multiple histone isoform variants that can exert structural changes at the atomic level on the functional properties of the nucleosome: 1 example is histone H2A.z, an H2A variant associated with active transcription, which operates by altering the interfaces between H2A–H2B dimers, as well as among the entire tetramer, partially destabilizing the nucleosome core (thereby facilitating nucleosome eviction during transcription).^3^ Another example is centromeric histone H3, called CENP-A, which replaces H3 in centromeric nucleosomes, altering the interactions among histones but maintaining the octameric structure.^4^ Mammalian genomes encode multiple versions of core histones, with each family hosting multiple variants of unknown significance. These histone isoform variants have been shown to participate in development and disease through altered roles in DNA replication, chromosome packaging, and DNA accessibility.^5,6^ Proteomics experiments have demonstrated variable expression of these isoforms in different cell types, including in the heart,^7^ and the stoichiometry of histones changes with disease, such as in pressure overload hypertrophy.^8^ In general, however, the nucleosome is among the most conserved protein structures known.

Where nucleosomes bind along the genome is influenced—certainly in reconstituted systems but likely also in vivo to some degree—by primary DNA sequence, with poly dA:dT–rich regions being comparably depleted of nucleosomes, due in part to the biophysical properties of these regions that resist bending necessary for nucleosome binding.^9^ An additional class of chromatin structural element includes non-nucleosome chromatin structural proteins, such as histone H1 family proteins, CTCF (CCCTC-binding factor), and HMG (high mobility group) proteins. Histone H1, the so-called linker histone (also known as histone H5), is not part of the nucleosome core particle but may participate in the formation of higher order chromatin fibers through interactions with nucleosome histones and DNA.^10^ Structures of nucleosomes with H1 (which has been termed the chromatosome^11^) show the latter nestled just outside of the core,^12,13^ making independent contacts with DNA, but whether linker histones are obligatory components of a structural feature in eukaryotic chromatin remains a matter of debate. Observations from a variety of eukaryotic systems implicate linker histone H1 in genomic functions, including DNA repair/stability, replication, and transcription.^14^ CTCF and HMG proteins each have likewise been attributed a wide range of possible functions, based mostly on gain-/loss-of-function studies in cell systems. As a general mechanism, CTCF binds DNA and facilitates chromatin looping (ie, the formation of semistable long range intrachromosomal interactions, spanning kilobases)^15^; specificity may arise in different cell types to facilitate regulatory interactions. HMG proteins, of which there are many families, bind and stabilize distinct structural features in DNA, thereby contributing to high order chromatin anatomy and perhaps assemblies of nucleosomes. Interestingly, the HMGA family of these proteins has been implicated in cardiac disease, with both hetero- and homozygous knockout mice developing hypertrophy.^16^

How nucleosomes position along chromatin is largely specified, in multicellular eukaryotes, by ATP-dependent chromatin remodeling enzymes and the trio of protein classes responsible for the so-called the histone code, those being writers, erasers, and readers, that add, remove, or interpret histone modifications, respectively. Although all these processes exist in other organelles and compartments (indeed many purported histone modifiers in fact interface with nonhistone, non-nuclear proteins), the allegorical grammar terminology adopted for chromatin post-translational modification (PTMs) has been particularly helpful in conceptualizing experiments into how gene expression is regulated and particularly effective in biasing the interpretation of results.

**Figure 1.**
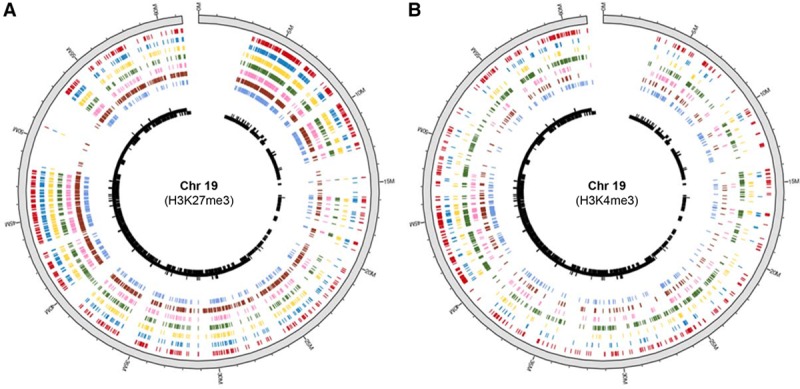
**Occupancy of histone marks varies between cell types.** Circos plot of mouse chr19 shows (**A**) repressive H3K27me3 and (**B**) active H3K4me3 peaks across different tissues. Color-coding of tissues (outside to inside): heart (red), cerebellum (blue), kidney (yellow), liver (green), thymus (orange), testis (dark red), and spleen (light blue). The black track represents mm10 gene density on chromosome 19. Data are from the ENCODE (Encyclopedia of DNA Elements) database.

**Figure 2.**
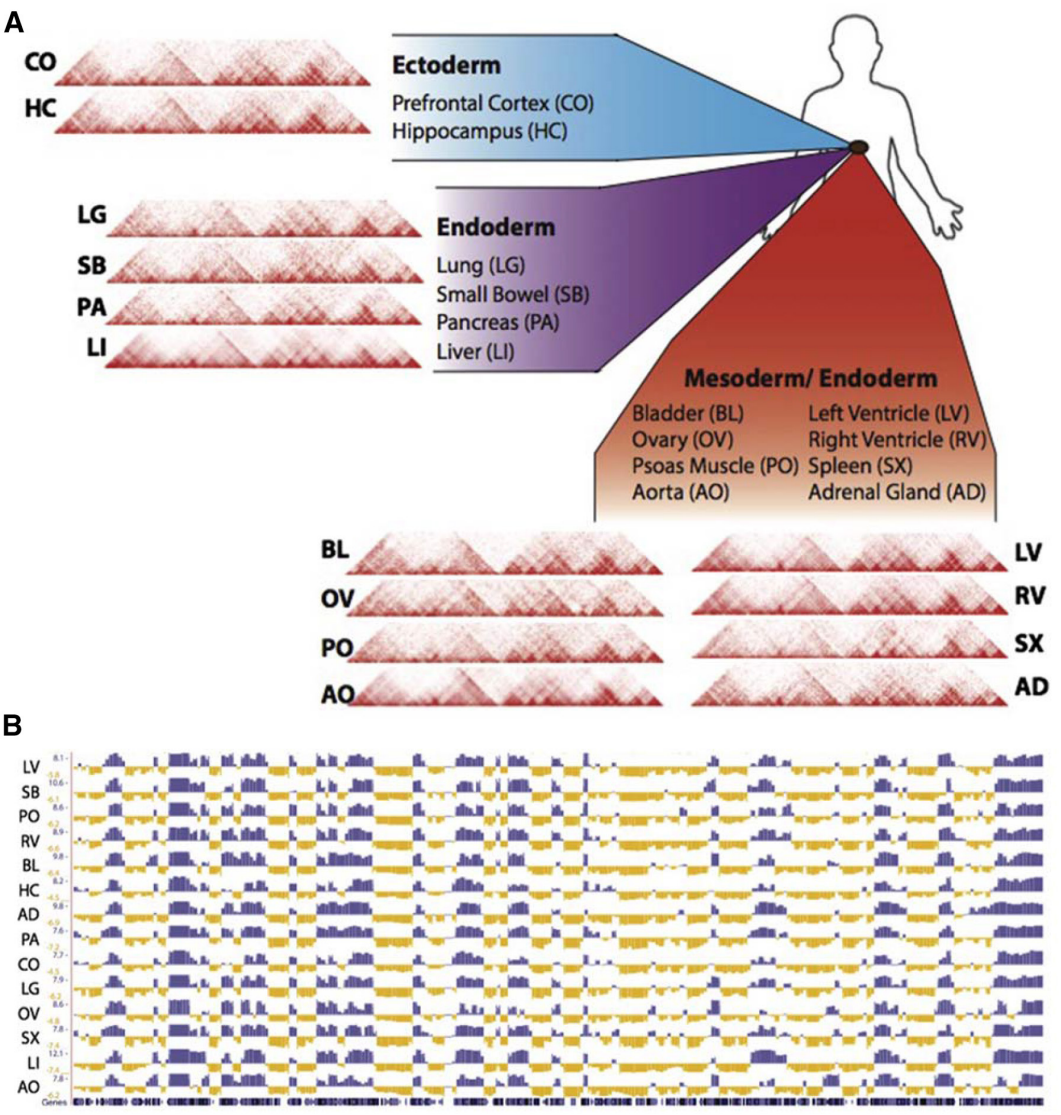
**Chromatin conformation capture data reveal similarities in chromatin organization between cell types. A**, Representative contact matrices from 14 human tissues (**A**), and their A/B compartmentalization profiles (**B**), demonstrate similarities at the scale of topologically associated domains (TADs) and compartmentalization. AD indicates adrenal gland; AO, aorta; BL, bladder; CO, prefrontal cortex; HC, hippocampus; LG, lung; LI, liver; LV, left ventricle; OV, ovary; PA, pancreas; PO, psoas muscle; RV, right ventricle; SB, small bowel; and SX, spleen. Black track represents mm10 gene density on chromosome 19. Data are from the ENCODE (Encyclopedia of DNA Elements) database. Adapted from Schmitt et al^21^ with permission. Copyright ©2016, the Authors.

Since their discovery and association with transcription or its inhibition, histone modifications have been the source of innumerable question-begging experiments and seemingly endless rounds of debate on their relationship to gene regulation.^17–19^ Histone post-translational modifications were originally described in the 1960s^20^ but only with the advent of chromatin immunoprecipitation and DNA sequencing (ChIP-seq; Figures 1 and 2) studies could the occupancy of these modifications be correlated, in a genome-wide manner in a single experiment, with gene transcription. Histone H3 has been particularly well characterized and shown to exhibit conserved regulatory behavior across species and cell type, with consensus existing about the roles of H3K27me3 in reversible gene silencing, H3K27ac in gene activation, H3K4me3 in gene activation, H3K9me3 in more lasting gene silencing and heterochromatin, although other histone isoforms, such as residue K20me on histone H4 (associated with gene silencing), exhibit evolutionarily conserved relationships to gene regulation. Histone modifications are associated with a variety of processes not related directly to transcription, including replication, DNA repair, mitosis, and cell division,^5,6,22^ although these processes tend to be better understood in model organisms like yeast and not extensively studied in mammalian systems in vivo.

The histone code hypothesis (discussed in greater detail below), as originally articulated by Strahl and Allis,^17^ states that a prescribed transcriptional or other genomic regulatory response arises, or is evoked, from a certain combination of histone PTMs in vivo. This hypothesis has tacitly established a framework in which to examine large amounts of ChIP-seq data, inspecting for regions of correlation between histone marks and gene expression. The resolution of most ChIP-seq peaks (≈200–1000 bp) is highly dependent on the informatics tools used to map reads and subsequently call peaks^23^ (because protein occupancy on the genome makes a much smaller footprint, it is consequently often unknown exactly where a protein binds; the less commonly applied ChIP-exo^24^ and X-ChIP-seq,^25^ achieving ≈25 to 50 bp resolution, are exceptions). Thus, one does not know whether histone PTMs occupy the same nucleosome, which would be required for a histone PTM reader protein to distinguish a full, accurate code from a partial and inaccurate code (example of partial exception: chromatin digestion to single nucleosomes coupled with mass spectrometry proved coexistence of modifications on the same particle [in an antibody-independent manner], which incidentally revealed that bivalency can occur at the single nucleosome level [ie, different histone H3 copies in the same complex have opposing—that is one has an active and the other a repressive—PTMs], so-called asymmetrically modified nucleosomes^26^).

Histone writers and erasers are signal transduction processes that sometimes use histones as substrates. The concept of the reader, however, is an indispensable component of epigenetic language and one without obvious counterpart outside the context of the chromatin (scaffolding proteins in signaling networks are a similar but distinct concept). To work as advertised—that is, to distinguish target region α from nontarget β, to bind α, and then to do something—readers must be able to simultaneously recognize and discriminate between histone PTMs, which has been shown in some model systems,^27^ but has not been demonstrated to operate in mammals in a tissue-specific manner. Progress has recently been made in the area of heart failure, however, with the example of the BET (bromodomain and extra-terminal) bromodomain protein BRD4 (bromodomain-containing protein 4), whose inhibition with the small molecule JQ1 prevents hypertrophy and associated transcriptional changes^28,29^ and whose pharmacological inhibition reverses some of the fibrotic and deleterious structural remodeling in the wake of infarction or pressure overload (having no effect on physiological hypertrophy).^30^ These studies have raised the intriguing possibility that epigenetic readers may be novel targets of epigenetic therapy in cardiovascular disease^31^ given their cell type–specific expression patterns, ability to integrate the actions of multiple chromatin modifiers, and their role to program gene expression with high fidelity at individual loci.

All 4 core histones are extensively post-translationally modified, with different histones (and different modifications) studied to varying extents and degrees of rigor and functional depth. The bane of accurate and reproducible proteomic mass spectrometry is that a truly unbiased experiment to detect PTMs will identify many, with no certainty about which ones are important versus otiose signaling noise. The most common answer to the question of which PTMs are important for chromatin regulation is some variation on “only the ones shown to be functionally involved in a phenotype.” Often the PTMs deemed most tantalizing are those previously published on in lower organisms and those for which commercially available antibodies exist. In the chromatin world, these PTMs and the associated antibodies are the ones getting the most game time in ChIP-seq experiments. A quantitative example: the silencing mark histone H3 lysine 27 trimethylation, which enriches around gene regulatory regions, is represented at the time of this writing by 403 ChIP-seq data sets in ENCODE (Encyclopedia of DNA Elements) and ≈2000 to 3000 publications in PubMed (an exact count of publications is tough given cavalier nomenclature); those numbers for H3 K27 acetylation, a euchromatic mark (note: on the same residue of the same protein), were 384 ChIP-seq datasets and ≈500 publications. In contrast, a recently identified succinylation on human histone H3 K79, also found to enrich in the transcription start sites of active genes, has 1 publication and 1 ChIP-seq data set.^32^ When examining a new PTM, no good antibody, and no tractable cell system for transgenesis of a tagged surrogate, means no genome-wide address book, which means, for the present, the given modification is assumed to be noise. That histone modifications cannot be unequivocally examined by genetic approaches (ie, by mutating a residue in the gene and studying the protein) is the reason why they are alternatively so frustrating and interesting.

What about other changes in protein levels in the nucleus? The first attempts to understand proteomic remodeling of the cardiomyopathic nucleus were conducted in hamsters.^33^ These early studies did not directly measure histone PTMs—although they (the PTMs) likely were reflected in the electrophoretic patterns reported in these investigations—because of the absence of accurate and quantitative mass spectrometry of proteins and PTMs. More recently, quantitative analysis of nuclear proteins^34^ or chromatin-associated proteins in the heart revealed the proteins regulating cardiac epigenomes^7^ and described changes in histone protein isoform stoichiometry^8^ in the setting of pressure overload hypertrophy, yet these studies did not characterize histone PTMs. Renal nuclear proteomes have also been explored,^36^ with physiological implications for cardiovascular disease. Across eukaryotes, ≈500 post-translational individual modifications have been identified just on the core nucleosome particle.^22^ Few of these have been identified, much less quantified and proficiently interrogated, in the cardiovascular system (most studies, to our knowledge, of histone PTM in the cardiovascular system rely on commercially available antibodies). Recent studies have used large libraries of histone modifications, in some cases representing >100 different nucleosome combinations (note: these are discrete, tailored nucleosomes with known histone post-translational modifications, in contrast to the total list of histone modifications, for which it is rarely known whether the modifications are happening on the same nucleosome, let alone in the same copy of the histone, with the exception of proteomic studies on intact proteins^37^), to investigate the ability of particular histone PTM combinations to influence the activity of human ATP-dependent chromatin remodelers.^38^ These libraries^39^ allow for the effects of individual recombinant nucleosomes with designer histone modifications (and combinations of modifications) to be tested in vitro for their ability to influence a host of chromatin properties, such as the binding preferences for transcription factors or chromatin-modifying enzymes.

## Role of Histone-Modifying Enzymes in Cardiovascular Physiology and Pathophysiology

Investigations into the enzymes responsible for depositing and removing histone modifications have demonstrated striking phenotypes in a range of cardiovascular syndromes. Histone deacetylases (HDACs) are one of the most extensively studied families of histone-modifying enzymes in the cardiovascular system. HDACs consist of 4 families, each with distinct isoforms which in turn have distinct histone and—in some cases—nonhistone targets, distinct cellular locations and distinct biological functions. Inhibition of HDACs has been shown pharmacologically (eg, with trichostatin A or valproic acid) to prevent proliferation of vascular smooth muscle cells^40,41^ (with implications for atherosclerosis^42^), to attenuate hypertension,^43^ to ameliorate ischemic/reperfusion injury and postischemic remodeling,^44–47^ and to block cardiac hypertrophy in the setting of heart failure.^48–50^ Molecular dissection of these phenomena, particularly in the setting of cardiac growth, have revealed HDACs to be powerful hypertrophic modulators: loss of HDAC9 leads to prodigious cardiac growth,^51^ HDAC4 and 5 regulate CamKII (calcium/calmodulin-dependent protein kinase II)-dependent gene regulation,^52,53^ HDAC2 regulates GSK3β (glycogen synthase kinase 3 beta)-Akt–dependent fetal gene activation in hypertrophy,^54^ to name just a few examples. Indeed, the literature on the role of HDAC targeting by drugs or genetic manipulation in the cardiovascular system is sufficiently vast to devour entire, authoritative reviews articles.^42,48,55,56^

Class III HDACs are also known as sirtuins and have been shown to exert powerful effects in the cardiovascular system after their initial identification and association with longevity in yeast. There are 7 sirtuin isoforms, each with varied subcellular localization and some of which, such as sirtuins 1, 2, 6, and 7, localize to nucleus and thus are poised to use DNA-bound histones as substrates, including in the heart.^57^ Multiple sirtuin isoforms have been shown to facilitate DNA repair through homologous recombination. Unlike other HDACs, sirtuins require nicotinamide adenine dinucleotide as a cofactor for activity. Intriguingly, these proteins have been shown to participate in an isoform-specific manner in myocardial ischemia, oxidative stress, apoptotic cell death, and cardiac hypertrophy.^57^ Sirtuin isoforms have also been shown to exert salubrious effects on fatty acid, amino acid, and glucose metabolism in mouse studies, which have led to clinical trials.^58^ A search at clinicaltrials.gov with the keywords cardiovascular disease and epigenetics revealed 29 studies in the active or recently completed phase. These studies range in their employment of epigenetic measurements or interventions. For example, some describe measuring epigenetic end points in response to treatments targeting known cardiovascular risk factors in common cardiovascular disease (eg, Epigenetic Reprogramming of Monocytes in Patients With Coronary Atherosclerosis, which measures epigenetic marks in the promoters of proinflammatory cytokines and chemokines in monocytes). Another example is a trial targeting rare genetic diseases with epigenetic manifestations in the cardiovascular system (X-chromosome Inactivation, Epigenetics and the Transcriptome), which measured DNA methylation, histone PTMs, and coding/noncoding RNAs expression in blood, white cells, and other tissues. Other examples investigate use of hypomethylating agents, including 5′azacytidine and 5-aza-2′-deoxycytidine, in adult patients with acute myeloid leukemia and atrial fibrillation (Action of the Vidaza on the Atrial Fibrillation) or study DNA methylation as a marker for high blood pressure in the setting of pregnancy (Observational Study of Epigenomic Dysregulation in Preeclampsia-Associated Chronic Hypertension). The Human Epigenetic Drug Database (hedds.org) is a useful, searchable resource for information on epigenetic drugs, their targets, and associated data sets including, when relevant, links to clinical trials. Several epigenetic drugs are Food and Drug Administration approved for clinical use, including HDAC inhibitors and DNA methyltransferase (DNMT) inhibitors,^59^ and several of these have been explored in cardiovascular disease (this review^60^ also includes an extensive summary of microRNAs targeted for therapeutic manipulation by pharmaceutical companies).

Histone acetyltransferases also constitute a large family of genes and have numerous nuclear and non-nuclear substrates (with type A being nuclear and type B being non-nuclear, mainly cytoplasmic) and have been characterized with varying degrees of specificity in cardiac and vascular cells.^56,61,62^ One of the best characterized histone acetyltransferases is p300 which, in addition to its common residence at enhancer elements, has been shown to regulate genes that inhibit endothelial cell inflammation in the setting of atherosclerosis^63^ and to attenuate salt-induced hypertensive heart failure^64^ and agonist-induced cardiac hypertrophy^65^ (the males absent on the first histone acetyltransferases has similar antihypertrophic actions when overexpressed in the mouse^66^).

The histone methyltransferase SET domain containing 2 was recently shown to be essential for myoblast differentiation in a process involving modification of histone H3K36 trimethylation.^67^ SET and MYND containing histone methyltransferase (Smyd; a family with 5 isoforms of varying tissue expressivity) family member Smyd1, which is restricted to striated muscle, has been shown to participate in cardiac phenotype through loss-of-function studies in the adult heart, which resulted in hypertrophy, dilation, and derepression of some cardiac disease genes.^68^ Although Smyd1 localizes to the nucleus and interacts with chromatin in the adult heart^68^ (and has been shown to regulate H3K4me3, an activating mark, in reconstituted systems^70^; mice with Smyd1 depletion in adulthood exhibited sustained H3K4me3 levels^68^; however, supporting the existence of alternative substrates for Smyd1 and indicating the existence of alternative proteins capable of maintaining H3K4me3 in cardiac myocytes), a substantial portion of the protein is non-nuclear. This non-nuclear population of the protein has also been implicated in adult cardiac function (along with another family member Smyd2^71^) and heart development.^72–74^ A different SET family protein, G9a (also known as euchromatic histone lysine methyltransferase 2), has been shown by loss-of-function studies to play a key role in adult cardiac phenotype: inducible MerCreMer-dependent depletion resulted in cardiac hypertrophy, modestly depressed ejection fraction, and fibrosis through a mechanism that involves targeted derangement of multiple histone methylation marks (including H3K9me2/3 and H3K27me3) at genes involved in cardiac function.^75^ SET7 was shown in a human microvascular endothelial cell line to regulate target gene (IL-8 [interleukin 8] and HMOX1 [heme oxygenase 1]) expression in a H3K4me1-dependent and -independent manner.^76^ Intriguingly, this process is tightly linked to glucose levels, demonstrating a metabolic sensing mechanism on chromatin.^77^ On the removal side of methylation, genetic loss or augmentation of the histone demethylase JMJD2A, respectively, blocked or exacerbated cardiac hypertrophy in mice.^78^ A recent pharmacological study showed that inhibition of histone methylation at H3K9 with the compound chaetocin (which targets the enzyme [SU(VAR)3–9] responsible for conversion of H3K9me2 to H3K9me3) attenuates some aspects of salt-induced cardiac dysfunction (survival rate, fractional shortening, and fibrosis)—while not affecting pathological gene regulation and only modestly impacting hypertrophic growth—in part through diminished H3K9me3 at repetitive elements and mitochondrial genes.^79^

The polycomb repressive complex (PRC) is one of the best-studied gene silencing complexes (responsible for H3K27me3 deposition) and has been implicated—usually through the actions and genetic disruption of one or more of its components—in a wide variety of higher phenotypes in mammals. In the cardiovascular system, the Ezh subunits 1 and 2 were shown to be differentially involved in cardiac development and regeneration: both were necessary for normal development, with Ezh1 but not 2 being required for neonatal heart regeneration and with Ezh1 but not 2 being capable, via overexpression, of promoting regeneration in the hearts of mice aged outside the established neonatal regenerative period,^80^ suggesting that features of myocyte proliferative/regenerative plasticity may be revived through histone-modifying enzymes. Ezh2 stabilizes forming blood vessels in the mouse embryo,^81^ and pharmacological inhibition of Ezh2 (and some H3K27me3 target loci) improved outcomes in hindlimb ischemia.^82^ Some naturally occurring compounds target histone-modulating enzymes (and nonhistone lysine residue-containing proteins) and have substantial in vivo benefit in conditions, such as cancer.^83,84^ Because many of these compounds are present in diets shown epidemiologically to promote cardiovascular health (such as cruciferous vegetables), part of this effect may be through actions to promote—at the subcellular level and across organs—favorable epigenomic health.

Although the histone isoforms and even specific residues targeted by individual enzymes have often been worked out in reconstituted systems in vitro, such information is almost universally lacking from such studies in animal models of the cardiovascular system (this observation is also true, incidentally, for most studies of acetylating/deacetylating and methylating/demethylating enzymes in noncardiovascular systems when examined at the organ level in animal models). It is also important to note that many of these histone-modifying enzymes target nonhistone substrates that have no direct relationship to gene expression or epigenetics but yet exhibit powerful effects on complex organ level phenotypes (cardiovascular examples include Smyd on titin,^71^ HDACs on myofilaments,^85^ and HDAC family members of the sirtuin class which regulate many substrates in mitochondria and cytosol^57^).

This leads to a couple interesting questions about chromatin-modifying enzymes in the cardiovascular system: First, what are the principles that allow for coordination of the various writers, readers, and erasers in the given cell type at any time (by this it is meant: how do the histone modifiers themselves get turned on or off, up or down, and when turned on, how do they compete for influence over gene expression in a reproducible manner?). And second, how do the cadre of expressed-at-any-given-time enzymes decide which nucleosomes to modify (ie, how is targeting accomplished, because most histone-modifying enzymes do not have DNA sequence-targeting motifs)? One approach to answer these questions would be to identify intermediate indices of epigenomic function like accessibility and structure, designing interventions that modulate these indices.

## ATP-Dependent Remodeling of Chromatin: Inducing and Participating in Cardiovascular Diseases

ATP-dependent chromatin remodeling enzyme complexes use the energy from ATP to translocate DNA through the nucleosome. That is, the ATP-dependent chromatin remodelers reposition nucleosomes along the genome according, in part, to cues harbored in the spectrum of histone tail PTMs, thereby enabling fundamental genomic processes, like transcription, nucleosome assembly/disassembly, mitosis, meiosis, and chromosome segregation. One of the most studied of these complexes is the SWItch/sucrose nonfermentable complex, originally identified in yeast (and known to have >10 protein components) and its mammalian cousin the brahma-associated factor complex (itself composed of >10 protein components, some of which exhibit tissue-specific expression). Models for the actions of these remodelers are informed by protein crystallography studies, in vitro biochemical assays, and ChIP-seq–based genome-wide measurements and are thus highly developed and can help to explain the observed dynamism of chromatin in development, between cell types and in stimulus response.^86–88^ Because presence of bivalent marks at a given locus are necessary but not sufficient to specify a bivalent locus, recent studies have focused on evaluating the role of ATP-dependent chromatin remodelers in this process.^89^ Studies in the cardiovascular system have examined the role of these complexes in tissue level phenotypes by genetic manipulation of the ATPase subunits of the brahma-associated factor complex, Brm (Brahma), or Brg1 (brahma-related gene 1, a.k.a. Smarca4). For example, genetic disruption of either of these molecules alone had no effect on retinal angiogenesis in neonates, exercise-induced angiogenesis in adult skeletal muscle, or tumor angiogenesis, whereas mice with disruption of both Brm and Brg1 after birth exhibited fatal vascular malfunction in the heart and gut during the early postnatal period^90^ (similar context-dependent functional redundancy, and lack thereof, was observed between Brm and Brg1 in the vascular endothelium, wherein disruption of both proteins in endothelial cells was required to observe tissue level defects^91^). Brg1 has been shown to be involved in zebrafish myocyte proliferation and cardiac regeneration,^92^ mesoderm, and hence cardiomyocyte, differentiation in cell culture, in part by modulating enhancer activity,^93^ whereas both Brg1 and Smarca3 (a.k.a. brahma-associated factor 60c, another brahma-associated factor complex member) are required for normal heart development in mouse^94–96^ (incidentally, Brg1 was found to be downregulated in adult murine hearts and re-expressed, concomitant with the myosin heavy chain isoform switch [α to β] associated with cardiac pathology; blocking Brg1 upregulation in the adult prevented this molecular event and attenuated hypertrophy^95^). Early formation of vasculature and erythropoiesis in mouse is dependent on Brg1 but not Brm in hematopoietic and endothelial cells,^97^ implying functional distinction between complexes seeded with these different ATPases during cardiovascular development (a similar conclusion was made from genetic disruption in smooth muscle cells^98^), a functional involvement that may extend into adulthood in the setting of endothelial injury and presumably disease.^99^

## Multifunctional Role of DNA Methylation in Chromatin Biology and Cardiovascular Phenotypes

DNA methylation is dynamic during vertebrate development, where it reinforces cell fate decisions and controls imprinting, or the dependence of gene/protein expression (and associated phenotypes) on whether a given version of a gene is expressed from maternal or paternal allele. Unlike histone modifications, which can occur on any number of different amino acids apparently without heed to locale (ie, without clear DNA consensus motifs), DNA methylation targets a single residue, cytosine, usually in a single context (ie, when followed by a guanine, so called CpG dinucleotides; recent evidence suggests, however, that this too may be an oversimplification as non-CpG DNA methylation, so-called CpH methylation [where the H connotes A, T, or C] occurs in some cells,^100^ such as neurons [where it may account for 25% of methylation], although this has only begun to be explored in the cardiovascular system^101,102^). Also contrasting with the plethora of proteins controlling histone PTM, DNA methylation is directly added or removed by a narrow suite of enzymes: maintenance (DNMT1) and de novo (DNMT3a and DNMT3b; DNMT2 modifies RNA and DNMTL is a catalytically inactive regulatory component of the methylation machinery) methyltransferases which establish methylation patterns after mitosis and replication and alter the pattern of methylation during organismal development and disease, respectively. Demethylation of DNA occurs in part via nonenzymatic means during replication, as well as during normal and pathological conditions in nondividing cells. Conversion of 5-methyl-cytosine to 5-hydroxymethylcytosine is catalyzed by the 10-11 translocation methylcytosine dioxygenase 1 family of enzymes—an active, selective process. The 5-hydroxymethylcytosine is a less stable modification, prone to nonenzymatic conversion to (unmodified) cytosine, and has thus been proposed as a molecular beacon of genes switching from off to on. DNA methylation patterns are erased and re-established transgenerationally, but this process seems to involve faithful perpetuation of methylation marks along the genetic lineage, that is, from parent to progeny (further evidence of this phenomenon can be seen in inbred mouse strains, whose DNA methylation landscapes are epigenetically preserved within a genetic lineage while being stably distinct between lineages^103,104^).

Bisulfite sequencing is the method for unequivocal determination of methylation status (note: all methods for DNA methylation analysis are in symbiosis with suites of informatics tools^105^). Broadly construed, DNA methylation in CpG islands (regions of genome with high frequencies of the dinucleotide, which incline toward promoters) and shores (areas around said islands) tends to be associated with gene silencing.^106^ Conversely, the bodies of mRNA-encoding genes tend to be methylated, without an established correlation allowing prediction of expression. Other methods for large scale analysis of DNA methylation include methylation immunoprecipitation (which has been used to identify methylation-dependent regulation of atherosclerotic risk in humans^107^) and DNA methylation arrays (notably the Illumina 450 chips), the latter of which has been extensively deployed in humans to characterize methylation patterns associated with a host of pathophysiological conditions including cancer,^108^ high blood pressure,^109^ body mass index and obesity,^110,111^ atrial fibrillation,^112^ inflammation,^113^ and death.^114^ Perhaps, because of cost and technical demands, reduced representational bisulfite sequencing (or much more expensive whole genome bisulfite sequencing) has been applied to a smaller list of diseases. Such data from mice, however, show that DNA methylation plays a powerful role in heritable differences in response to metabolic syndrome^103^ and may contribute to catecholamine-induced cardiac pathology.^104^ DNA methylation and hydroxymethylation abnormalities have been found in animal^115,116^ and human^117,118^ heart failure, associated with changes in expression of pathological genes. Work from mouse cardiomyocytes suggests that DNA methylation largely obeys chromatin structural features of A/B compartmentalization (itself defined based on gene density, histone marks, and other features of open chromatin; see section below on chromatin structure), wherein dynamics of DNA methylation during lineage commitment are enriched in A (active) compartments and genetic disruption of DNA methylation (via DNMT3a and 3b knockout) does not alter compartmentalization.^102^ This observation supports a passive relationship between DNA methylation and chromatin structure, at least in the formation phase.

What is the import that methylation patterns are associated with complex human phenotypes? One method through which DNA methylation has its molecular effects is to reinforce prevailing chromatin landscapes by preventing accessibility and facilitating compaction (in promoters, as mentioned above, and in X chromosome inactivation, where it conspires with histone H3K27me3 to silence expression of 1 of the 2 X chromosomes in females^119,120^); another is to favor relaxing of chromatin and transcription (as in gene bodies). These opposing effects must require the intervention of discriminating factor(s), perhaps including methyl-CpG binding proteins, but this field currently wants for established rules and actors. One investigation^121^ demonstrated that MeCP2, a methyl-CpG binding protein, is reversibly downregulated in a mouse model of pressure overload (when the aortic banding was removed, MeCP2 expression was restored; similar observations in patients with LVADs [left ventricular assist device] suggest this process may be operative in humans). These findings suggest that the actions of DNA methylation may be modulated in the diseased heart at multiple levels, including methylation, demethylation, and reading of methylation. Another mode of action is through *trans* effects, whereby a methylation event can regulate the expression of a gene in a distal region of the genome (ie, far away from the actual CpG in question). Studies from human cardiac development reveal an enrichment of regulatory elements, including DNA methylation sites, in regions of genetic variation associated with heart disease,^122^ supporting a molecular link between chromatin regulation and genetic variation in the context of pathological phenotypes. By regulate, it is meant here that the methylation event is shown to correlate—with genome-wide statistical significance—with the expression of a gene, a methylation quantitative trait locus. This regulation may take the form of enhancer element formation/modification (discussed below) or other as-yet uncharacterized chromatin structure effect.

The prevalence of cancer^123^ and congenital heart disease^124^ in humans is associated with mutations in genes encoding proteins that modify chromatin, such as histone-modifying enzymes (writers, erasers, and readers). Furthermore, these complex diseases are often associated with global changes in DNA methylation. In some cases, the genetic or epigenetic lesion occurs in a gene whose aberrant function can exert a dominant role in disease pathogenesis. In other cases, these epigenomic changes may instead be general hallmarks of perturbed cellular function, whereby the normal parameter space for gene expression is expanded, facilitating dysfunction of multiple cellular processes. For many observations on histone PTMs and DNA methylation in cardiovascular disease, the train or snow leopard question remains unanswered.

## Role of Noncoding RNAs in Chromatin Function and Cardiovascular Physiology

It is now appreciated that most of the genome is transcribed, if only a small portion of that transcriptome encodes mRNA destined for translation, with intriguing differences in this noncoding transcriptome across cell types and after pathological insult. Noncoding RNA biology is a specialized discipline unto itself, with new species of RNA—ascribed really cool and sometimes bizarre functions—identified seemingly endlessly and will not be extensively reviewed herein (excellent reviews on the roles of various noncoding RNAs in cardiovascular biology have emerged^125–128^). Of particular interest to chromatin biology, however, is the concept that long noncoding RNAs (lncRNAs) may participate in gene regulation by modulating chromatin structure.

lncRNAs have been proposed as a potential mechanism for how different chromatin marks are deposited—specifically and reproducibly—across the genome. One of the best-studied lncRNAs, a general definition of which is an RNA >200 nucleotides with no discernable open reading frames (an exception to this being the presence in some lncRNAs of open reading frames which have been shown to produce micropeptides that go on to regulate key intracellular processes in cardiovascular cells^129,130^), is *Xist*, which is centrally involved in X chromosome inactivation. *Xist* is transcribed from and acts in *cis* to silence the X chromosome through a process that recruits, via direct binding of *Xist* to the proteins, the PRC2 complex, and YY1. A depositor of histone H3K37me3 silencing marks and transcriptional repressor, respectively, these proteins in turn compact the X chromosome and prevent further transcription.^120,131^ This model—lncRNA binding to a specific region of chromatin and recruiting histone-modifying enzymes—is appealing because it solves the problem of DNA sequence recognition, of which many histone modifiers are incapable. Another well-characterized lncRNA that binds PRC2 subunits is *Hotair*, involved in gene silencing in mammals and shown to regulate chromatin in *trans* outside of the context of X inactivation.^132^ These studies led to a gold rush on PRC2-interacting lncRNAs, which have been estimated to range in number from hundreds to thousands in mice^133^ and humans.^134^ The general properties, if they exist, through which these lncRNAs couple PRC2 to chromatin are the focus of continued investigation.^135,136^ It may be that the genes for these molecules are distributed across the genome and the lncRNAs in turn all act in a local manner to recruit and modulate chromatin machinery to a given gene expression environment.^137^ Yet there are clear limitations were the cell to attempt to repeat this process with other lncRNAs: physiological transcriptional profiles in adult cells do not involve turning on or off entire chromosomes, with genes temporally coregulated often residing on different chromosomes (each of which, in this model, would require its own lncRNA, although 3-dimensional chromatin environments may allow transcription factories to form bringing multiple mRNA-coding genes into a neighborhood governed by a single lncRNA) and beset by numerous histone modifications. Reflecting this fact, the spectrum of lncRNA functions has expanded^120,138^ to include actions in *trans* (ie, targeting other chromosomes) as well as *cis* to enhance transcription, to block it, to scaffold chromatin interactions, and to aggregate microRNAs (thereby making them unavailable to regulate mRNAs).

Initial investigations of lncRNAs in the heart revealed involvement in developmental growth and maturation. *Fendrr* binds both PRC2 and the activating complex Trithorax group/MLL in mesoderm, its depletion leading to impaired cardiac and chest wall development.^139^ *Braveheart*, another mesoderm-associated lncRNA, binds the Suz12 subunit of PRC2 and is required for proper differentiation of embryonic stem cells into cardiac precursors.^140^ Also involved in cardiac development is the lncRNA Upperhand that regulates the *Hand 2* locus in *cis* by facilitating chromatin modifications (super enhancer maintenance) and RNA pol II elongation.^141^ Other lncRNAs have been discovered to play a role in disease-associated gene regulation. *Chaer* binds the Ezh2 subunit of PRC2, and its genetic manipulation leads to alteration in H3K27me3 levels around pathological genes and cardiac hypertrophy in the mouse.^142^ An antisense transcript in the β-MHC (beta-myosin heavy chain) locus was found to associate with that locus in a manner independent of the PRC subunit EZH2 in the setting of pressure overload.^143^ Interestingly, that same paper showed the EZH2 interaction with chromatin was regulated by the noncoding RNA pri-miR-208b, hinting at a broader role for noncoding RNAs in regulating chromatin.^128^ Some lncRNAs seem to exert their effects on gene regulation through interaction with chromatin remodeling complexes, as is the case for *Mantis*, a lncRNA discovered in macaque and shown to regulate endothelial angiogenesis in a manner involving interaction with BRG1.^144^ Similarly, the cardiac-specific lncRNA *Myheart* binds and inhibits the actions of BRG1, thereby regulating expression of myosin heavy chain expression, along with other genes. *Myheart* is downregulated by pressure overload stress and its transgenic restoration protects against overload-induced hypertrophy.^145^ Interestingly, lncRNAs like *Malat1* in vascular tissues^146^ and *Chast* in cardiac tissues^147^ are nuclear localized and regulate expression of nearby genes although it remains to be tested whether they accomplish these actions through recruitment of chromatin complexes. In the field of cholesterol metabolism, 2 recent lncRNAs have been discovered that exert powerful effects of lipid levels and atherosclerosis in vivo: *LeXis*,^148^ expressed in the liver, directly controls genes involved in cholesterol biosynthesis, consequently modulating plasma cholesterol levels, and *MeXis*,^149^ expressed in macrophages, regulates genes involved in cholesterol efflux (both lncRNAs were found to operate through chromatin based on subcellular localization, accessibility assays, and transcriptional regulation).

Discovery analyses in a mouse model of pressure overload revealed the expression profile of cardiac lncRNAs,^150^ determining their extent of enrichment in this tissue when compared with tissues of distinct developmental origin (liver and skin) and determining changes between embryonic, adult, and diseased noncoding transcriptomes (nota bene: only a few of the developmentally silenced lncRNAs were re-expressed with disease, in contrast to the fetal gene program^151^ documented for mRNAs). Likewise in myocardial infarction, the recovery/injury/remodeling period was found, in mice, to be associated with changes in lncRNA expression (incidentally, the lncRNAs were also found to reside near chromatin marks associated with transcriptional enhancement), some of which (the lncRNAs) were subsequently shown to modulate expression of mRNA-encoding genes known to participate in basic cardiac function.^152^ Studies from humans have charted differences in lncRNA expression between fetal and adult cardiomyocytes, linking their expression with known enhancer marks associated with protein-coding RNA transcription (eg, H3K4 methylation).^153^

What is known about lncRNAs in the cardiovascular system is that they can be cell type specific, often lack extensive sequence conservation across species (although they may be conserved at the level of secondary structure), can regulate transcription (probably mostly in *cis*), and can correlate with histone PTMs, binding some of the histone-modifying complexes, PRC2 in particular. It is unknown to what extent lncRNAs can act at a distance (beyond, say, a few kilobases from their own site of transcription), the role of chromatin structure to coordinate such actions (evidence from X inactivation suggests that local chromatin environment—rather than DNA consensus motifs—facilitates *Xist* binding, PRC recruitment, and inactivating activity^131^), if they bind directly to chromatin and DNA (perhaps involving triple helix formation^154,155^), and whether they are sufficient to coordinate the locus-specific activities of chromatin modifiers through a model in which multiple lncRNA genes, by virtue of their evolutionary distribution at key sites across the genome, establish local neighborhoods of regulation at which they recruit—or repel, according to wont—histone modifiers and polymerase machinery.

## Cardiovascular Development- and Disease-Associated Enhancer Elements

Enhancers are regions of DNA that promote the transcription of other regions of DNA.^156–158^ A contemporary synthesis on how this works: specific histone PTMs (eg, histone H3K4me1 and H3K27ac; some histone isoforms, such as H3.3 and H2az, contribute to enhancer activity; enhancers are now thus commonly identified by genome-wide ChIP-seq experiments) decorate regions of DNA that need not be—although may be (see below)—themselves transcribed, which in turn recruit binding of enhancer-associated proteins (eg, lineage relevant transcription factors, RNA pol II, and coactivator proteins, such as p300 and Mediator) and interact in 3 dimensions with the genes whose expression they enhance. This region of DNA, the appropriately demarcated histones, and any associated proteins together constitute the enhancer which is often validated as such by showing that either (1) its genetic disruption interferes with expression of its target gene or (2) that the enhancer DNA sequence can drive developmental and lineage appropriate transcription through a cell- or organism-based reporter assay. In the absence of chromatin conformation data, enhancers are usually assumed (and tested) to regulate the nearest downstream gene. Somewhat counterintuitively, then, enhancers tend to reside in areas of relative nucleosome depletion (not, strictly speaking, in areas devoid of nucleosomes), such that enhancers can be identified by open chromatin assays (eg, DNAse I hypersensitivity or ATAC) followed by DNA sequencing. A subgroup of enhancers, called super enhancers,^159^ has been classified based on the observation that the aforementioned enhancer features at times occur multiple times in close proximity to each other. Super enhancers can exhibit augmented transcriptional activation potential and thus may represent a distinct structural property of cell type–specific chromatin.^160^ Further specification of enhancer behavior includes delineation of poised (those ready for promoting transcription of their targets) versus active (those actually so promoting) enhancers, which can be distinguished by the presence of silencing histone marks (and the enzymes that deposit them) at poised enhancers and their absence (concomitant with the presence of greater levels of RNA pol II) at active enhancers.^156–158^

It has more recently become apparent that some enhancers may themselves be transcribed^161^ and may thus operate in the RNA form. It could be that this transcription is a goal-directed process in the normal way we think about RNA doing things in the cell: the enhancer RNAs may have gene regulatory or other functions. It may also be that the enhancer RNA synthesis is a by-product of enhancer DNA in close apposition to churning transcription factories and serves no subsequent end and that its transcription serves the end of keeping a transcription factory churning and poised for ready enlistment in production of other RNAs that do serve subsequent ends (as RNAs). This fascinating concept of chromatin biology is an active area of investigation.^162^ Studies have begun to emerge examining the role of enhancer transcription in select cardiovascular processes, such as cardiac conduction^163^ and endothelial cell stress response.^164^ Active endothelial cell enhancers, defined by H3K4me2 and H3K27ac binding (plus some enhancer RNA transcription), exhibited altered transcription factor binding in human aortic endothelial cells after exposure to oxidized phospholipids.^164^ Intriguingly, SNPs (single nucleotide polymorphisms) associated with cardiovascular disease were over represented in these enhancers, suggesting a molecular scale explanation for how the former influences transcription and phenotype, a property that may be a common feature of enhancers across cell types and species.^165^

p300 occupancy has been used to identify enhancers in the developing mouse heart (embryonic day 11.5), many of which were found to exhibit tissue-specific activity.^166^ A similar approach was used to characterize enhancers in fetal and adult human heart tissue (of note, 48% of the enhancers were the same in fetal and adult human hearts; when comparing fetal mouse to fetal human, the overlap was 21%),^167^ revealing functional elements that may participate in human cardiac gene regulation. Angiotensin II–induced vascular growth, a key component of atherosclerosis, was found to proceed via dynamic utilization of super enhancers in human cells through a process that involves complex interplay among noncoding RNAs, chromatin readers, and transcriptional machinery.^168^ A theme of developmental processes being redeployed—not wholesale, but in a selective manner—in the disease setting may also play out for enhancers: regulatory elements marked by H3K27ac are specified in part by the master cardiac transcription factor GATA4 during development and some of these regions, devoid of GATA4 in healthy adult heart, are revisited by the protein on pathological stimulation, contributing to disease-associated gene expression.^169^ Pursuing this concept more directly in a complementary model, it has also been shown that cardiac enhancers undergo altered regulation by disease-associated transcription factors after pathological stress.^170^ The histone modification reader BRD4 binds super enhancers that are associated with cardiac disease genes. Interestingly, this process is finely tuned to differentially modulate association of BRD4 with these disease genes while leaving housekeeping genes unaffected, a process controlled in part by microRNA-dependent titration of BRD4 levels.^171^

## Unexpected Cell Type–Specific Functions of Chromatin

The rapidly dividing phenotype of cancer has allowed researchers in this field to identify epigenetic clones^172^: lineages of cells outwardly genetically identical that differ based on semistable, transmissible chromatin features. All cardiac myocytes and vascular smooth muscle cells are not the same, which means that although these cells do not proliferate and differentiate like cancerous cells do, it is reasonable to hypothesize that developmentally endowed epigenetic clones exist and contribute to organ level phenotypes in the adult. Indeed, distinct clonal populations (arising from a common progenitor in development, rather than from a resident adult stem cell) of cardiac cells contribute to different anatomic and functional features of the adult organ^173–178^ (recent single cell studies have revealed these distinct myocyte populations to indeed exhibit distinct transcriptomes^179,180^)—epigenetic dissection of these populations may well reveal epigenetic clonality to be an underlying process contributing to this observation. The chromatin accessibility assay ATAC-seq, which reveals areas of open chromatin, has been applied in a single-cell format to a lymphoblastoid cell line, identifying subpopulations of cells based on chromatin accessibility.^181^ That such variability exists in postmitotic, healthy adult cells in the cardiovascular system remains to be demonstrated, but the observation of transcriptome variability in these cells, and the presence of chromatin accessibility variability in cells otherwise phenotypically similar, makes such a conjecture not unwarranted.

Chromatin can act like a stress sensor complex, wherein there is no single factor controlling changes in disease-associated gene expression. Some investigators have described excitation–transcription coupling, with the term specifically applied to local calcium signals around the nucleus (as distinguished from global calcium transients involved in myofilament contraction) inducing local CaMKII activation and HDAC mobilization.^182^ What if this observation is evidence of a more generalized, myocyte-specific sensory apparatus on chromatin, that detects local calcium signaling, such as that involved in pathological gene activation, from calcium involved in contraction and nonetheless critical to influence gene expression (eg, sustained faster heart rates require greater turnover of proteins and thus transcripts)? Various pathological cell states, including cardiovascular disease and cancer, have been characterized by global changes (eg, that revealed by a total cell lysate Western blot or genome-wide ChIP-seq signal) in histone modification. One possible reason for this unexpected observation was found to include regulation of cellular acidity^183^: global histone acetylation responds to perturbation of cellular pH (lower pH leads to less histone acetylation) and cells respond to modulation of histone acetylation by modulating pH, a sort of acetate capacitance system on chromatin to attenuate large swings in cellular acidity. Combined metabolomic and proteomic studies reveal that abundance of short chain acyl-CoA donors directly, although not indiscriminately, influences the modification of histone tails in human cells in culture.^184^ Indeed metabolic sensing by chromatin has been increasingly recognized to underpin cardiovascular physiology and disease.^77^

Aberrations in nuclear rigidity and structural integrity are associated with diseases like cancer and progeria, some of which are driven by so-called laminopathies, arising from malfunction of nuclear lamina proteins. Cardiomyopathies resulting from mutations in lamin A/C are one of the best-studied group of genetic diseases in clinical cardiology and have led to clinical trials, although in this context the effects on chromatin structure are unclear. Aberrant nuclear morphology, the blebbing of the nuclear membrane because of impaired laminar network architecture, is a hallmark of these diseases.^185^ Association of chromatin with the lamina seems to be essential for nuclear structure, and disrupting this interaction has detrimental effects on nuclear integrity,^186^ particularly in cells subject to mechanical force. These actions are coupled to the mechanisms known to regulate chromatin’s role in gene expression, as supported by the observation that histone PTM influences nuclear rigidity and membrane integrity.^187^

An unexpected non-nuclear, signaling behavior of not just chromatin-modifying proteins but actual intact multimolecular slabs of chromatin has been observed in cancer^188^: cytoplasmic chromatin fragments—evaginated nuclear membrane containing DNA and nucleosomes decorated with heterochromatin marks—can induce inflammation and cell death through cytoplasmic signaling and circle-back transcriptional regulation. An even weirder story: rod and cone cells are terminally differentiated, specialized components of the retina, the light-sensitive component of the vertebrate eye. Evolution has hijacked chromatin in rods (but not cones) to serve the transcriptionally unrelated function of focusing light in the retinas of nocturnal but not diurnal mammals (ie, the organization of DNA in the nucleus forms a physical lens),^189^ thereby providing a meta-function in service of that specific cell’s raison d’être.

**Figure 3.**
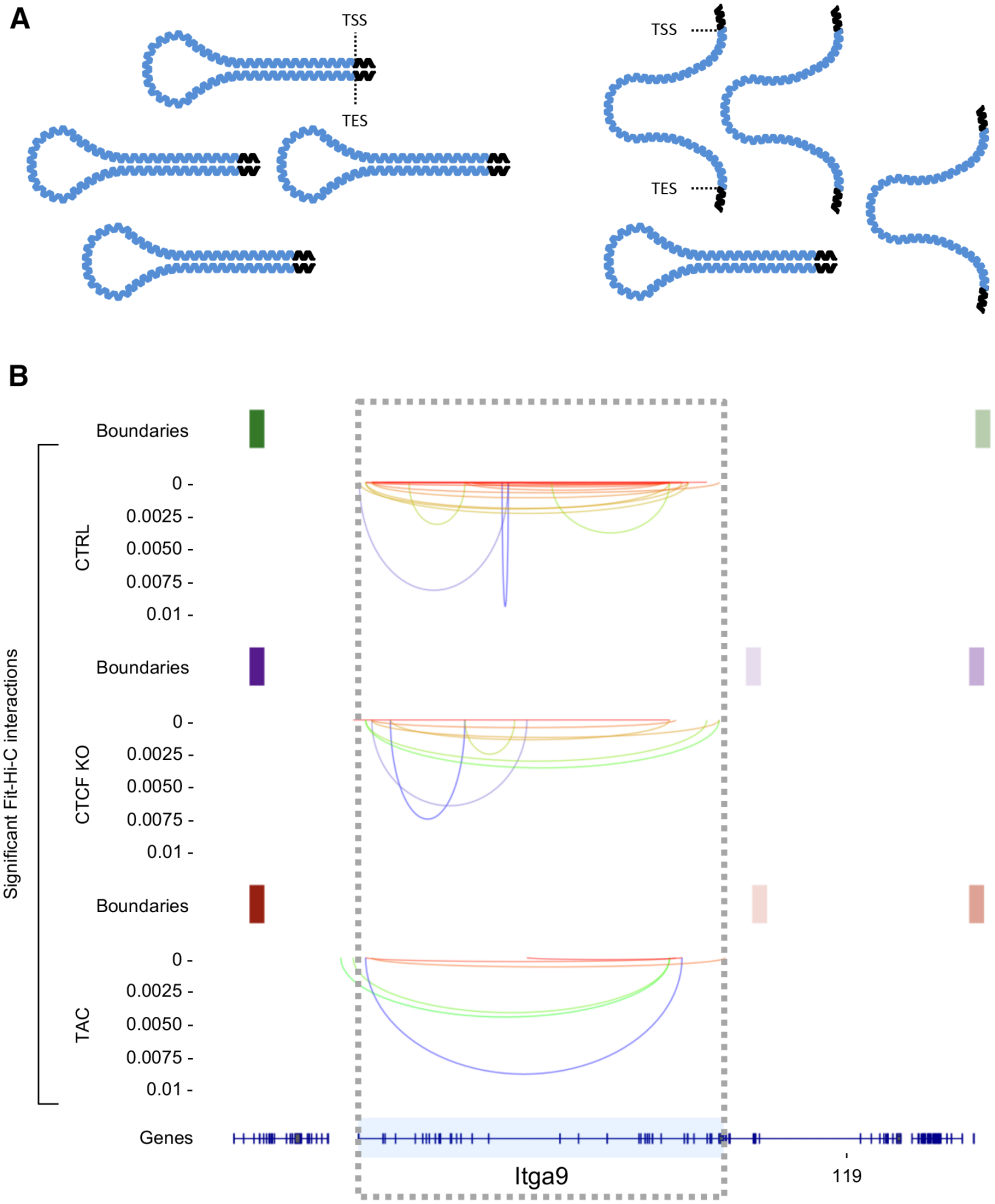
**Chromatin looping. A**, Schematic representation of chromatin looping, in this example between transcription start (TSS) and end (TES) sites of a gene. The model is an interpretation of chromatin capture data (which shows a decrease in interactions during cardiac pathology) and is intended to represent the frequency of a given conformation, not a population effect across cells: **left**, under normal conditions, loops are stably formed; **right**, because loops are less stable, they are less frequently captured experimentally. **B**, An example gene displaying this behavior. Top is control, middle is CTCF (CCCTC-binding factor) knockout, and bottom is transverse aortic constriction. Rectangles are topologically associated domain boundaries, and lines are chromatin interactions detected by Hi-C (Reprinted from Rosa-Garrido et al^191^ with permission. Copyright ©2017, the American Heart Association). Online Table II shows a list of genes that undergo the phenomenon described in the figure.

Different structural units of chromatin establish distinct transcriptional environments. It can be helpful to think of chromatin itself as a transcription factor or transcriptional processor. Transcription does not happen willy-nilly throughout the nucleus but rather is localized to transcription factories,^190^ or areas designated to different forms of transcription, such as rRNAs and house-keeping sorts of protein-coding mRNAs, separated from stimulus responsive genes and furthermore from transcriptionally silent regions. This happens on a nuclear scale as reflected by the observation that transcription tends to happen toward the center of the nucleus whereas the periphery is an area of gene silencing. Another type of transcription factor-like activity is subchromosomal, in the form of chromatin looping, the formation of short- and long-range interactions to facilitate gene activation (ie, enhancer elements) or repression (ie, insulators or boundary elements). Chromatin capture data have revealed that long range looping within the epigenome is dynamic and can bring together transcription start and end sites in 3 dimensions, perhaps to facilitate efficient cycling of machinery like polymerases and transcription factors (Hi-C data support this concept for a cohort of genes, Figure 3; Online Table II). This principle has been supported with ChIP-seq data from rat hearts,^192^ in which different transcriptional activation profiles (pause-release and de novo recruitment) have been described in the setting of pressure overload hypertrophy, along with accumulation of RNA pol II in transcription end site, perhaps reflective of gene looping.

## Structure-Function Features of Chromatin and Implications for Cardiovascular Gene Regulation

**Figure 4.**
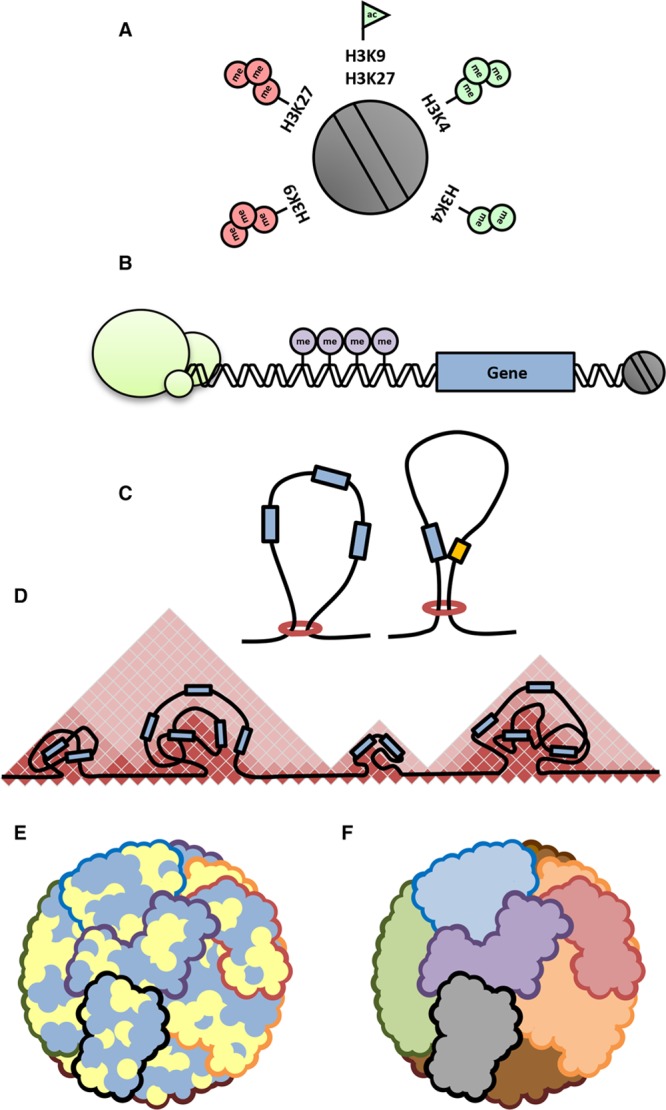
**Chromatin architectural features. A**, The functional unit of chromatin is the nucleosome, which can be decorated by a variety of post-translational modifications that modulate accessibility to transcription factors or chromatin modifiers. **B**, At the gene level, transcription factors or repressors (green circles) confer context-specific regulation of transcription with varying levels of sequence specificity. DNA methylation (purple circles) typically repress promoter activity of genes although this phenomenon is associated with expression when found within gene bodies. **C**, Chromatin looping enables formation of gene expression or silencing neighborhoods, as well as facilitating structural units suitable for higher order packing. **D**, Topologically associating domains (TADs) are regions of preferential chromatin interactions. **E**, Hi-C data reveal chromatin compartmentalization into active and inactive, or A and B compartments of the genome, respectively (here shown in yellow and blue; note, this is a stylistic interpretation of how A and B compartments might interact because chromatin capture studies do not reveal actual localization coordinates within the nucleus). **F**, Chromosome paint experiments have revealed distinct territories that contain entire chromosomes within the nucleus, allowing formation of intra- and interchromosomal interactions that may regulate transcription or other tasks of the nucleus.

Based on insights from chromatin capture and other epigenomic techniques, the organization of the epigenome is thought to involve key structural intermediates (Figure 4). What is the evidence that chromatin is inherently ordered above the level of the nucleosome (where data exists to the atomic—that is several angstrom—level^2^) and below the level of the chromosome (where chromosome painting, closer to the scale of micrometers, demonstrates compartmentalization^193^)? The goal of chromatin structural studies is to determine: what are the structural features between these scales and at what scale(s) are structural features functionally important? Next consider the pattern of chromatin observed in various cells of the cardiovascular lineage with a quotidian method, such as DAPI (4',6-diamidino-2phenylindole) labeling: while the pattern of staining is not random, there is no obvious reproducible pattern within a class of cells (and not shared between 2 classes) to which a functional consequence can be intuited (for a nifty exception, see ref ^189^), in contrast to chromosome patterns in mitosis/meiosis which definitively exhibit such tell-tale architecture. Fluorescence in situ hybridization experiments clearly demonstrated spatial segregation of chromosomes into territories^193^ while not revealing evidence of hierarchical arrangement. Recent higher resolution electron microscopy–based imaging of chromatin shows that its structure, in both interphase and mitotic cells, rarely achieves a scale >24 nanometers in diameter (for reference, the nucleosome diameter is ≈11 nm), with distinctions in arrangement between such cells coming from the density of compaction, which the authors interpret to be evidence of an absence of repeating, stable, hierarchical structure.^194^ How do these observations hold up in analyses of individual genes and with respect to histone post-translational modifications? In the cardiovascular arena, combination of Dam-ID and LaminB ChIP-seq (to identify loci associated with the nuclear periphery) and fluorescence in situ hybridization was used to demonstrate that differentiation in the myocyte lineage involves precise reorganization of expressed genes away from the myocyte nuclear membrane, itself found to be decorated with the silencing mark H3K9me2, and to a lesser degree by H3K9me3 (other silencing marks H3K27me2/3 and H4K20me2/3 were not found enriched at the periphery in skeletal myoblasts).^195^

A prediction of a nonhierarchical model of chromatin is that the size of structural elements should be normally distributed. For chromatin interactions detected by Hi-C, for example in cardiac myocytes, this prediction has been shown to be true: the number of interactions plotted per locus follows a normal distribution, where most locus bins (bin size=5kb) have the same number of interactions (≈2500) and a small number have very few or very many interactions (see Online Figure IA in reference ^191^). The vast majority of loci interact with a median number of other loci, and no privileged structural behavior can be assigned to the regions with large interactions (as would be a prediction of a scale free or hierarchical topology).

Additional insights from the explosion of chromatin capture techniques to determine endogenous interactions have been informative.^21,196^ These studies have characterized features of chromatin organization that are conserved across species and cell type (note: method development for analysis of chromatin capture data is ongoing and the interpretation evolves with it^197^): topologically associated domains (TADs) are regions of chromatin with privileged local interaction; TAD boundaries, as nominally implied, demarcate regions of the genome where intrachromosomal interactions switch from interacting in 1 direction (say 5′ biased) to the opposite; distinct regions are insulated against expression, in part by chromatin structural proteins and histone modifications; short and long range interactions reproducibly form (ie, the structure is not random). The boundaries represent epigenomic cornerstones, directing interactions of nearby DNA and proteins in alternating directions, in part through the binding of chromatin structural proteins like CTCF. Cohesin and CTCF knockout animals and cells indicate these proteins are involved in TAD maintenance,^191,198,199^ but these proteins alone are not the whole story: embryonic stem cells^198^ with only 4% normal CTCF protein levels still exhibited TADs and cardiomyocytes^191^ with 20% normal CTCF protein levels exhibited sparse, minor changes in TAD boundaries and strength. These studies also show that TAD formation/maintenance and A/B compartmentalization can be decoupled experimentally as loss of cohesin or CTCF did not affect A/B compartmentalization. A compartments have more genes and are defined by having less interactions than would be expected for a given distance (B has more), indicating less compact chromatin. Eu- and heterochromatin marks dominate in A and B compartments, respectively. Prevailing evidence suggests that at the level of TADs, chromatin architecture is quite similar between cell types. Sub-TAD interactions, and less abundant—yet reproducibly detected—long-range interactions that span multiple TADs (as well as interchromosomal interactions), may be the scales at which cell type–specific chromatin interactions are observed.

Chromatin structure is dynamic during the cell cycle. The predominance of longer range interactions present in postmitotic cells is rapidly lost upon entrance into G1, followed by further depletion throughout S and G2, in favor of shorter range, local interactions. This process abruptly reverses itself, with a return of TADs and long-range interactions after nuclear division.^200^ Mitotic chromosomes lack TADs and chromatin neighborhoods, instead exhibiting a uniform, homogenous pattern of hierarchical interactions.^201^ A similar observation was made for oocytes in metaphase II: an absence of TADs and chromatin neighborhoods in these cells persisted in the zygote, with long range chromatin interactions manifesting at the 8-cell and inner cell mass stages.^202^ Interestingly, physical segregation of alleles was seen to persist until the 8-cell stage as well,^202^ even after the formation of long range chromatin contacts, suggesting that chromatin structure is an emergent property of an allele, can vary between alleles, and thus may participate in allelic inheritance.

Probabilistic modeling has been used to reveal 3-dimensional organizational principles from Hi-C data sets, the goal here being not a structure per se, but a population-based representation of the structural features of the chromosomes as they associate in the nucleus.^203^ Using data sets from human lymphoblastoid cells, this approach was used to schematize genome structure, revealing interchromosomal surfaces of apposition and detecting new anatomic properties of the nucleus, such as the physical clustering of centromeres of different chromosomes and the anatomic positioning of euchromatin and heterochromatin pockets with respect to other nuclear landmarks. Unlike traditional fluorescence in situ hybridization or chromosome painting, in such an exercise one can know which loci are responsible for a given anatomic feature and in what part of the nucleus this feature tends to occur relative to other features.

The histone code^17^ has become a ready-to-hand tool for chromatin interrogation, shaping how studies are designed and interpreted. However, as discussed elsewhere in this essay, hundreds of histone PTMs are now known to exist. Also, the histone code and related ideas^204^ lack an underpinning in mathematical rules, a limitation addressed by investigators who have used dry laboratory approaches to characterize chromatin states^205^ or rules of nucleosome positioning^206^ that reconcile ChIP-seq and chromatin accessibility data with genome sequence and transcription. Apart from the accordant histone and chromatin-binding proteins associated with different flavors of chromatin, how distinct chromatin domains form, in a physical sense, is not completely understood. Recent evidence from Drosophila and human chromatin suggests that heterochromatin domains comprised H3K9me3-marked nucleosomes, heterochromatin protein 1, and DNA can exhibit phase separation behavior, which may be an explanation to link domain-scale and molecular-scale properties of heterochromatin foci which can display both liquid and stable phase properties,^207^ a phenomenon supported on a broader scale by contemporaneous studies.^160^ The way forward is to integrate structural studies from imaging and sequencings techniques with genome occupancy studies to build a new model governed by principles that incorporate all these sets of data.

## Integration by the Epigenome of Genetic Susceptibility and Environmental Risk

Histones were originally identified as inhibitors of transcription. This concept remains a kernel of chromatin theory: heterochromatin increases during differentiation and loss of pluripotency, and some diseases, notably cancer, have been found to be associated with a more euchromatic environment. Because they change concomitant with gene expression and phenotype, chromatin modifications are ispo facto taken as responsible for the unidirectional progression of cell fate commitment in the cardiovascular system. Another hypothesis cached therein is that histone PTM and other chromatin marks stabilize cell identity. Regarding this conclusion, here is a premise that should be rejected: identification of a chromatin-modifying enzyme in the heart or vasculature whose genetic manipulation impairs or reverses developmental state is a necessary and sufficient condition to prove a role for chromatin in deciding and stabilizing cell fate. Here is another such premise of tenuous utility: if chromatin modifications stabilize cell phenotype, then reprogramming strategies that restore pluripotency (eg, iPS [induced pluripotent stem]) or directly convert one cell type to another^208,209^ must do so by wholesale reprogramming of chromatin (although these processes do, no doubt about it, reprogram histone post-translational modifications and DNA methylation at cardiac genes^210^). iPS-derived cells coaxed toward a cardiovascular lineage acquire regulatory elements (ie, histone post-translational modifications on regulatory elements nearby lineage appropriate genes) reminiscent of their endogenous counterparts,^211^ and yet studies from noncardiovascular tissues have shown that iPS-derived cells retain some epigenetic memories from their cells of origin, which, perhaps not surprisingly, is also the case for cardiovascular cells derived in cell culture from developmental precursors (eg, DNA methylation).^212^ Cardiac cells exhibiting progenitor-like behavior isolated from adult hearts indeed exhibit, commensurate with transcriptome changes, DNA methylation changes in genes associated with the mature cardiomyocyte lineage, vis-à-vis adult cardiomyocytes lacking such progenitor-like behavior.^213^

**Figure 5.**
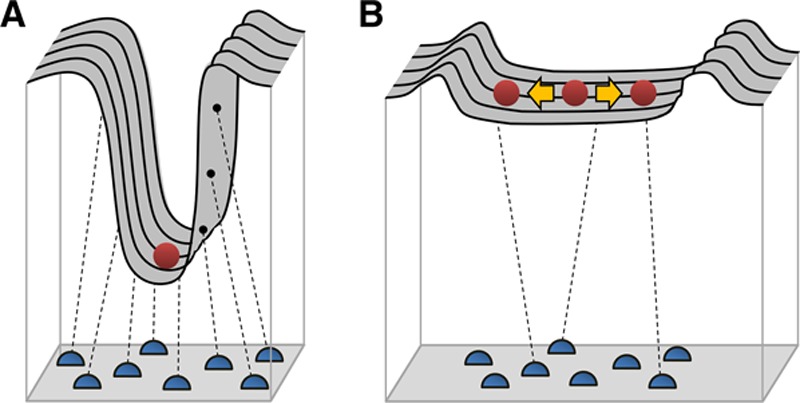
**Plasticity in epigenomic landscapes may allow for transcriptome reprogramming in disease.** Adapting Waddington’s concept of, to paraphrase, the chemical tendencies underpinning the epigenetic landscape (*The Strategy of the Genes*, New York: The Macmillan Company; 1957), the figure depicts how, when some of the chemical tendencies (**A**; which we now know to be the DNA, proteins, and RNA that establish the 3-dimensional structure of the epigenome, blue semicircles) are perturbed by experiment or environment (**B**), the red ball (which here represents a cell or cell population) can adopt different positions along the energy landscape, becoming sufficiently plastic to enable disease-associated gene expression.

**Figure 6.**
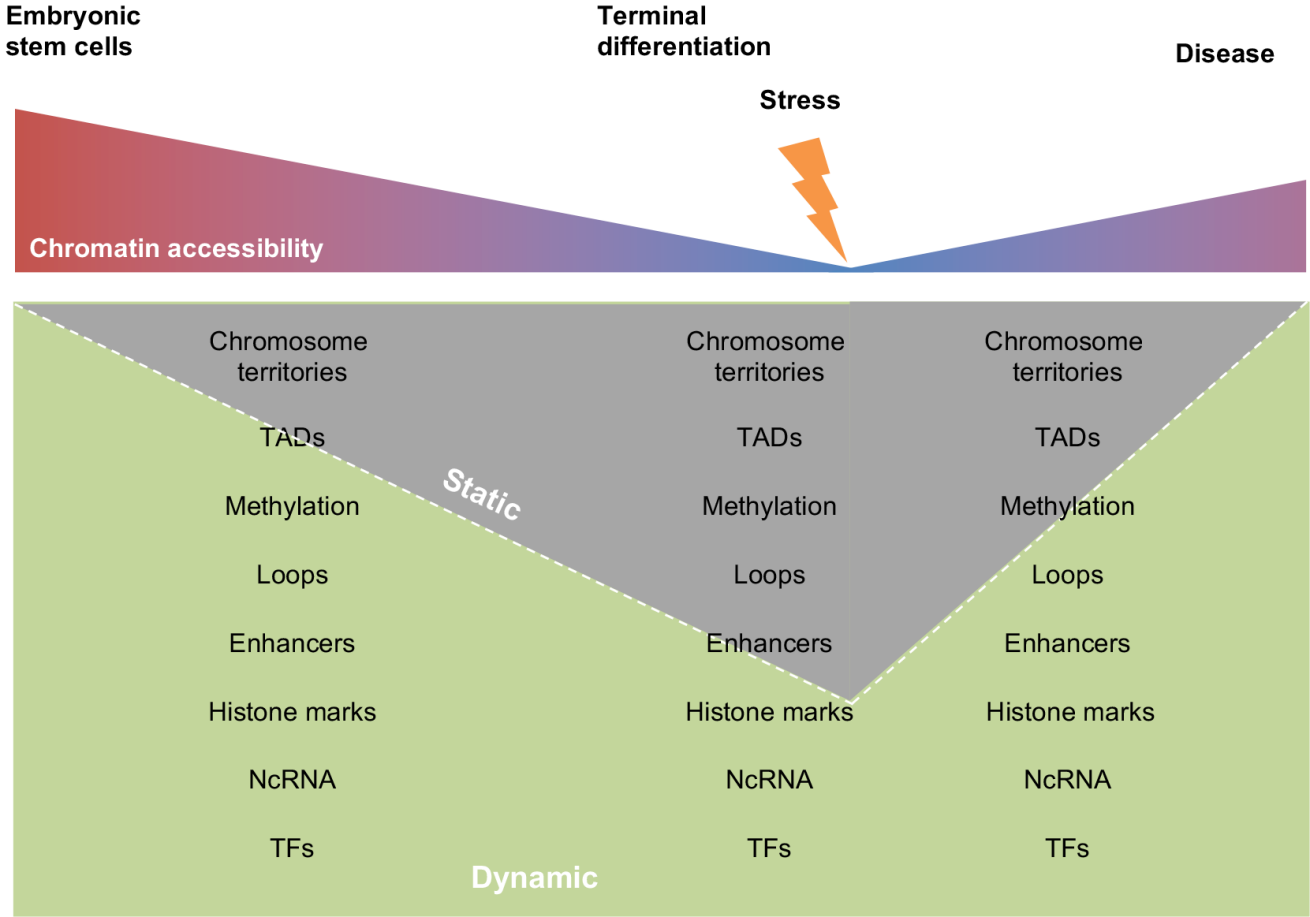
**Model for epigenomic changes in development and disease.** Development is accompanied by changes in chromatin structure and regulation to endow terminally differentiated cells with stable transcriptomes. Disease upsets this balance, transitioning select regions of the genome into more dynamic conformations through effects on chromatin structure, enhancer-gene looping and alterations in histone modification, DNA methylation, and other factors. This model is based on findings reviewed in the current paper and adapted from Rosa-Garrido et al^191^ with permission. Copyright ©2017, the American Heart Association.

Cell culture studies of distinct stages of cardiac lineage commitment explored the changes in chromatin marks associated with this process.^214,215^ General features of heterochromatic mark (H3K27me3) loss were observed around genes that were expressed (genes never expressed in the cardiac lineage, in contrast, retained abundant H3K27me3 through differentiation and never gained activating marks like H3K4me3), and genes that would be expressed in subsequent stages of development were sometimes (although not always) enriched with H3K4me1 (a so-called poised enhancer mark) before acquisition of H3K4me3 and RNA pol II concomitant with expression. If one were so inclined, the following observations may be taken as evidence that chromatin becomes more plastic in the setting of cardiac pathology (see also Figures 5 and 6): stimulation of neonatal rat ventricular myocytes with isoproterenol leads to decreased density of chromatin as measured by histone H3 immunolabeling and super resolution microscopy^216^; pressure overload hypertrophy is associated with a decrease in total histone H3K9me3 and increase in total H3K4me3 (as detected by Western blotting), as well as a decrease in the linker histone H1 to core (measured by H4) ratio^8^; association across genetically variable mouse strains between select chromatin structural proteins HMGB2 and CTCF and cardiac phenotype and the ability of HMGB2 to modulate cardiomyocyte hypertrophy and chromatin accessibility in cardiac myocytes^217^; and loss of CTCF (which induces cardiac dysfunction) or pressure overload hypertrophy is accompanied by a global decrease in genomic interactions detected by Hi-C.^191^

Meta-analysis of heart failure GWAS (Genome-Wide Association Study) studies recently uncovered a novel risk allele associated with mortality and located in a noncoding enhancer region.^218^ Interestingly, DNA methylation signatures at this locus in blood were correlated with allergic sensitization, potentially hinting at a gene–environment interaction leaving an epigenetic signature. A similar observation of epigenetic risk conferred in a cell type–specific manner by marks present across different tissues was recently reported in the context of dilated cardiomyopathy.^219^ The durability of these marks in a temporal sense remains an open question. Unequivocal determination of transgenerational inheritance of chromatin features like DNA methylation or histone modifications (discussion of which often conflates the 2 distinct concepts of epigenetics and Lamarckian evolution or the inheritance of acquired traits) is tricky,^220^ and many examples are hotly debated. Epigenome-wide association analyses in liver demonstrate^103^ DNA methylation–dependent—and sequence variation independent—associations with clinical traits important for cardiovascular disease, such as insulin level, as well as other omics end points, providing proof of concept for population level epigenomic regulation of complex disease traits through the actions of DNA methylation variation to control phenotype, presumably through effects on chromatin structure or accessibility. Something ostensibly heritable through cell division or meiosis may masquerade as epigenetic, and may even be of chromatic origin, while in fact proceeding via genetic means.^221^

**Figure 7.**
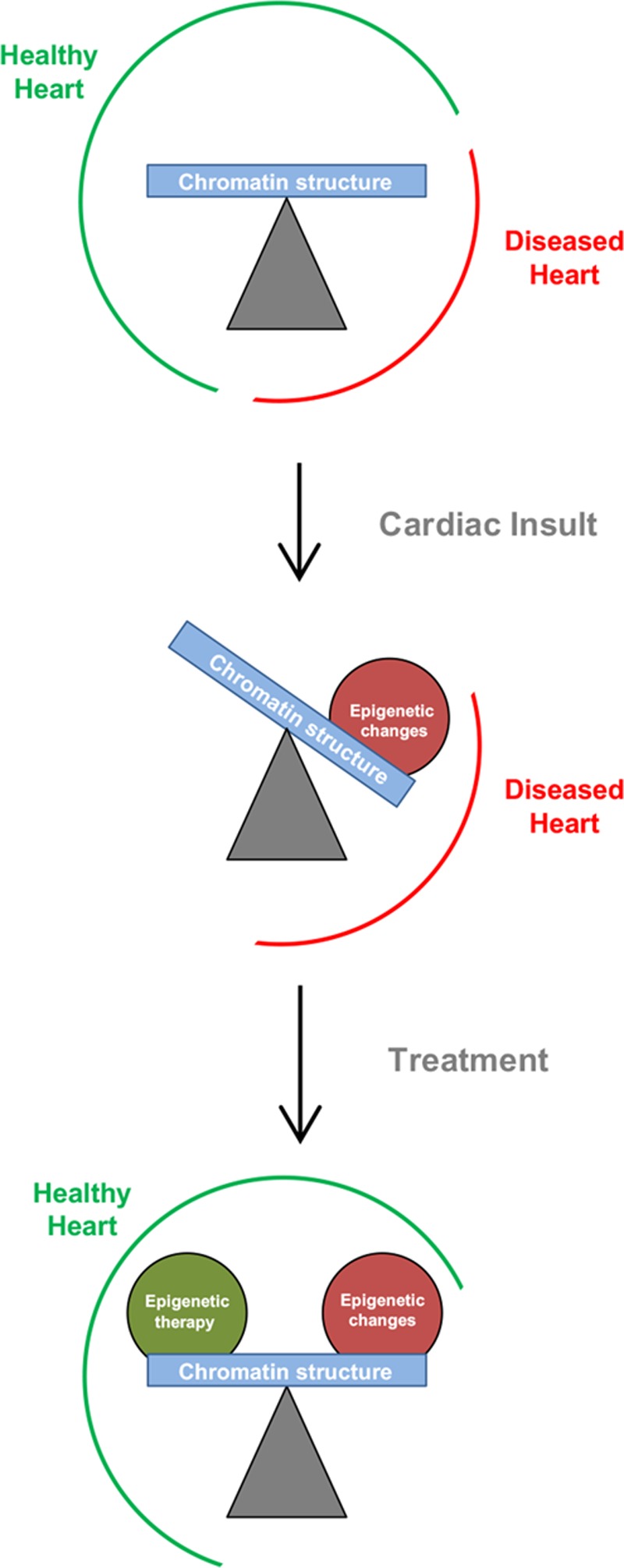
**Cardiac epigenetic therapies.** Changes in chromatin structure during cardiovascular pathologies alter gene expression and thereby phenotype. Epigenetic therapies (examples include histone deacetylases inhibition, BET (bromodomain and extra-terminal) inhibition, chromatin structural protein modulation, and chromatin loop or DNA methylation targeting by CRISPR/Cas9 tools) could be designed to reverse these changes by targeting intermediate chromatin features, such as accessibility and structure.

It has been increasingly recognized that epigenomic modifications are influenced by various diet and lifestyle factors that affect cardiovascular health (reviewed in detail elsewhere^222^). Maternal smoking, for example, is known to induce widespread DNA methylation differences in newborns, some of which persist into the offspring’s adulthood, including in genes known to be associated with smoking-related birth defects,^223^ although whether these effects are because of natal exposure remains unknown. There have been limited experimental studies directly testing the role of nongenetic inheritance of cardiovascular risk in animal models (eg, DNA methylation–dependent target gene expression in the context of offspring ischemic injury^224^). In vitro fertilization experiments in mice using gametes from obese or normal weight parents (note: obesity was induced by high-fat diet) and surrogate, normal chow fed, mothers revealed that a propensity for increased body weight can be inherited by nongenetic means.^225^ Metabolic gene regulation has been shown to be a modifiable, and subsequently heritable feature, in that mice fed low-protein diets passed hepatic gene expression profiles transgenerationally through the paternal germ line, commensurate with heritable changes in DNA methylation (although to what extent differences in DNA methylation resulting from distinct paternal diets directly control gene expression profiles remains unknown).^226^ Genetic variability contributes to chromatin accessibility (measured via FAIRE-seq) in the basal state and following complex metabolic changes, such as those accompanying high-fat diet.^227^ Human studies in ethnically diverse populations have revealed DNA methylation variation associated with nicotine and alcohol dependence (and the codependency between these forms of addiction)^228^ although no evidence of inheritance or precedence of the phenotypes by the epigenetic features was demonstrated. In addition to the obvious prognostic and diagnostic potential of genomic and epigenomic measurements in the clinical arena (the practical considerations of which are discussed in detail elsewhere^229,230^), it is noteworthy that tools for reducing epigenomic treatment to practice have begun to emerge (Figure 7). Modification of the CRISPR/Cas9 gene targeting system, involving an inactive Cas9 nuclease (so-called dCas9) fused to DNMT3a or Tet1 and combined with guide RNAs to localize the complex, can induce altered DNA methylation of specific loci in somatic cells in vivo commensurate with desired changes in gene expression^231^ (techniques for remodeling chromatin loops with designer CRISPR tools have also emerged^232^). Such approaches may enable targeting of entire transcriptomes, rather than individual molecules, in a gene therapy workflow that at once provides both specificity and temporal tuning.

Genomic and epigenomic technologies are providing high-resolution descriptions of susceptibilities and pathogenesis of cardiovascular diseases—that is, we now have the ability to acquire a far greater number of data points on patient populations, enabling identification of new biology but also, in the clinical setting, better stratification.^230^ The other omics technologies (such as proteomics, lipidomics, and metabolomics) that are now experimentally mature can, when applied to chromatin, provide greater still molecular detail on how transcriptomes are specified and cell type–specific behavior governed in health and disease. Effective utilization of this wealth of knowledge to promote human health will require innovative thinking: some strategies will involve multimarker panels (eg, DNA sequence variants measured along with protein or lipid levels to make diagnoses, such as, in hypertension) whereas other strategies will use the intermediate end points that emerge from the collective actions of multiple classes of molecules as the readout for diagnoses or as target for treatment (eg, targeting chromatin readers or chromatin accessibility, both of which integrate the actions of various signaling processes, protein modifiers, metabolites, and RNAs acting in the context of genetic variation).

It occurs to us that another definition of epigenomes would be the molecular features that make a living creature the same unit tomorrow that it is today. As described in the preceding sections, recent studies have identified the actions of discrete protein components and modifiers of chromatin in cardiovascular health and disease, providing potential targets for therapy. Epigenomic investigations have described chromatin landscapes, providing the data necessary to discover principles for the actions of the protein and RNA modifiers. These epigenomic investigations also enable discovery of intermediate properties, like chromatin accessibility and structure, which may be traits that contribute to higher level phenotypes. As the integrator of genetics and environment, and the substrate of cellular memory, chromatin features may provide the basis for understanding normal and pathological cardiovascular function.

## Acknowledgments

We regret that space limitations have forced us to omit reference to many important papers in the field of cardiovascular epigenomics. We thank Dr Emma Monte for critical feedback on this manuscript. Author contributions are as follows: concepts, all authors; text, T.M. Vondriska; figures, M. Rosa-Garrido and D.J. Chapski; and final version, all authors.

## Sources of Funding

Work in the Vondriska lab is supported by the National Institutes of Health, the American Heart Association, and the Cardiovascular Theme in the David Geffen School of Medicine at University of California, Los Angeles.

## Disclosures

None.

## Supplementary Material
