# Supplementary material for: Epigenomes in Cardiovascular Disease
Source: Circ Res. 2018 May 24;122(11):1586–607. doi: 10.1161/CIRCRESAHA.118.311597 (PMC5976233; doi:10.1161/CIRCRESAHA.118.311597)
Supplement: Supplementary file 1 [file res-122-1586-s001.pdf]

Family	Gene	Manipulation	Model	Phenotype	Molecular Targets	Reference (PMID)
Histone Methyltransferases	Ehmt2	Knockout	Mouse	Required for cardiomyocyte homeostasis<br>Its depletion causes cardiac hypertrophy	Required for the maintenance of heterochromatin that silence developmental genes in the adult heart	28778944
	Ezh1	Knockout	Mouse	Normal embryo.<br>Ezh1 and 2 double KO causes lethal heart malformations, including hypertrabeculation, compact myocardial hypoplasia, and ventricular septal defects. Ezh1 improves heart function and stimulated cardiac myocyte proliferation	Development and cell proliferation genes	28512107
	Ezh2	Cardiac specific knockout	Mouse	Normal embryo. Ezh1 and 2 double KO causes lethal heart malformations, including hypertrabeculation, compact myocardial hypoplasia, and ventricular septal defects	Development and cell proliferation genes	28512107
				Perinatal lethal; Double outlet right ventricle; Underdeveloped endocardial cushion due to decreased proliferation and increased apoptosis; Impaired septal formation, hypertrabeculation; Hypoplasia	Upregulation of negative cell cycle regulators (e.g. Ink4a, Ink4b), non-cardiac transcription factors (Pax6, Six1, Isl1), atrial and conduction gene expression in ventricles (Hcn4, Mlc2a);	22158708, 22312437
					Downregulation of Tbx2, Hey2 transcription factors	
		Knockout		Embryonic lethality with compromised vascular integrity and increased extracellular matrix degradation	Mmp9 and its activators Fosl1 and Klf5	25359725
	Kmtd2	Knockout	Mouse	Embryonic lethality<br>Conditional deletion at E10.5 shows developmental delay, pericardial edema and a linear heart tube	Essential for regulating cardiac gene expression during heart development via H3K4 di-methylation	26932671
	Mll	Knockdown	HUVEC Cells	Reduction in endothelial-cell migration and sprouting	HoxA9 and HoxD3	17047146
	Mll2	Knockout	Mouse	Embryonic lethal by E14.5<br>Apoptotic	-	16540515, 23826075
		Point mutation		Embryonic lethal by E11.5; Impaired looping		
	Setd2	Knockout	C2C12	Defects in myoblast differentiation and significantly reduction of histone 3 phosphorylation leading defects in cell proliferation	Myosin heavy chain (MHC) and Myogenin (MyoG) downregulation	28130125
	Smyd1	Knockdown	Zebrafish	Heterozygotes show no noticeable impairments	Upregulation of heat-shock proteins	16477022, 24068325
				Impaired cardiac function<br>Disrupted sarcomeric organization		
		Knockout	Mouse	Cellular hypertrophy, organ remodeling, and heart failure	Transcriptional repression of Tgfbeta3 and Nppa genes	27663768
	Whsc1	Knockout	Mouse	Lethal by P10 and growth defects<br>Impaired septal formation due to hypoplasia	Upregulation of Nkx2-5 dependent genes (e.g. Pdgfra, Isl1)	19483677
				Heterozygotes display some phenotypes of knockout due to haploinsufficiency		
Histone Demethylases	Jarid2/Jumonji	Knockout	Mouse	Perinatal lethal; Thin ventricular walls; Double-outlet right ventricle; Impaired septal formation, hypertrabeculation; Edema; Increased proliferation	Maintained fetal gene expression (increase in βMHC to αMHC ratio, ANF not repressed); Impaired patterning of cardiac gene expression (e.g. ventricular ANF expression maintained, high Mlc2a expression in ventricles and atria); Increase in Notch signaling	10807864
				Increase in mitotic activity in isolated cardiomyocytes	Upregulation of Cyclin D	15870077
				Perinatal lethal; Thin ventricular wall; Double-outlet right ventricle; Increase endocardium and myocardium spacing; Impaired septal formation, hypertrabeculation; Increased proliferation	Maintained fetal gene expression (no increase in αMHC, ANF not repressed); Increase in Notch signaling	21402699
	Jmjd2a	Gain and loss of function	Mouse	KO attenuates hypertrophic.<br>Overexpression exacerbates hypertrophy	FHL1	21555854
	Jmjd3	Knockout	mESC	Embryonic lethal by E6.5; Impaired *in vitro* mesoderm and endothelial differentiation in embryonic stem cells	Downregulation of mesoderm and cardiac markers (Brachyury, Mef2c, αMHC, Nppa)	23856522
	Jmjd6	Knockdown	mESC	Decreased in vitro network formation and sprouting in the spheroid assay during the endothelial differentiation of ESCs	Alters Flt1splicing increasing the levels of its soluble form which inhibits angiogenesis.	21300889
	Jmjd8	Knockdown	mESC	Decreased *in vitro* network formation and sprouting in the spheroid assay during the endothelial differentiation of ESCs	Jmjd8 controls glycolysis and respiration of ECs	27199445
	UTX	Knockout	Mouse	Embryonic stem cells cannot differentiate into beating cardiomyocyte *in vitro*	Repression of cardiac markers (ANF, α-cardiac actin, Myh6)	22192413
				Embryonic lethal by E10.5; Impaired looping, cardiomyocyte differentiation	Downregulation of cardiac genes (ANF, Mlc2v)	
Histone Deacetylase	Hdac1, Hdac2	Knockdown	Adult Mouse Ventricular Myocytes	--	Stimulates PAI-1 Expression	28174211
	Hdac6	Knockdown	Rat Cardiomyocytes	--	Upregulation of HDAC10 mRNA expression.	21539845
Protein Kinase	CK2α1	Overexpression	Mouse	Interventricular septum and the left ventricular wall thickness increase	α-actin or β-tubulin upregulation<br>HDAC2 phosphorylation	21576649
Transcriptional Regulation	Meis2	Knockdown	Zebrafish	Reduced heart rate<br>Pericardial edema indicative of cardiac failure	Genes involved in heart tube formation and cardiac looping	22981225
	TFIIB	Knockdown	Rat and Mouse Neonatal Cardiac Myocytes	Ameliorate pressure overload hypertrophy	Genes requiring de novo recruitment of pol II	25398966
	Yy1	Overexpression and Knocdown	Neonatal Mouse Ventricular Myocytes	Up-regulation of all genes present during fetal development and increases cell size	HDAC5	18632988

Supplemental Table I. Gain/Loss of Function Studies on Chromatin Modifiers in the Cardiovascular System.

Family	Gene	Manipulation	Model	Phenotype	Molecular Targets	Reference (PMID)
ATP-dependent Remodelers	Baf60c	Knockdown	Mouse	Embryonic lethal by E11; Impaired looping, trabeculation, short outflow tract; Hypoplasia	Differential patterns of cardiac gene expression (e.g. Nkx2-5, Nppa, Hand1, Hand2 affected)	15525990, 17210915
	Baf180	Knockout	Mouse	Embryonic lethal by E15.5; Reduced heart rate Impaired septal formation; Hypoplasia; Edema	Downregulation of cell growth and proliferation genes; Upregulation of growth arrest-associated genes	15601824
	Baf250a	Knockdown	P19 Cells	--	Upregulation of cardiac markers (e.g. Nkx2-5, Hand1, Acta2)	22621927, 24335282
		Overexpression		--	Downregulation of cardiac markers (e.g. cTnT, Nkx2-5, Gata4)	
		Knockout	Mouse	Embryonic lethal; Impaired trabeculation of right ventricle, no septum formation, outflow tract; Hypoplasia	Downregulation of cardiac transcription factors (Nkx2-5, Bmp10, Mef2c)	
	Brg1	Cardiac-specific knockout	Zebrafish	Embryonic lethal by E11.5; Impaired looping, no septum formation; Thin ventricular walls due to hypoplasia Embryonic lethal by E11.5; Double-outlet right ventricle; Decreased ventricular chamber size; Impaired looping, septal formation	Downregulation of Bmp1; Increase in αMHC to βMHC ratio Downregulation of cardiac genes (e.g. Nppa, Tbx5, Bmp10)	20596014
		Ventricle-specific knockout	Zebrafish	Neonatal heterozygotes show dilation chambers; impaired septal formation; impaired electrical function	Differential patterns of cardiac gene expression (e.g. Bmp4, Tbx2)	21304516
		Null mutation	Zebrafish	Arrhythmia Hypoplasia	Required for the activation of the Wt1 locus	28737171
		Vascular-specific mutations	Mouse	No evidence of defective angiogenesis. Brg1 and Brm double mutant die with multiorgan hemorrhage including small intestine and heart.	-	25904594
	Brm	Global mutation	Mouse	No evidence of defective angiogenesis. Brg1 and Brm double mutant die with multiorgan hemorrhage including small intestine and heart.	-	
	Chd4	Endothelial-specific knockout	Mouse	Embryonic lethal by E12.5; Impaired vascular integrity, reduced extracellular matrix, hemorrhaging; Hypotrabeculation	Downregulation of extracellular proteins (e.g. Type IV collagen, fibronectin); Differential expression of plasmin regulators to result in activation of matrix metalloproteases	24335282, 24348274
		Knockdown	Mouse	--	Upregulation of cardiac genes (e.g. cTnT, Bmp10, Gata4)	
	Chd7	Knockout	Mouse	Embryonic lethal by E10.5; Interrupted aortic arch	-	17334657, 19855134, 22363697
		Knockdown	Zebrafish	Impaired looping; Pericardial edema	-	
	Dpf3	Knockdown	Zebrafish	Impaired looping; Weak contractility Disrupted myofibril organization	Upregulation of genes involved in metabolic processes. Downregulation of genes involved in ion/electron transport and cell homeostasis	18765789
	Pontin	Knockdown	Zebrafish	Hyperplasia	-	12464178
	Reptin	Active mutant	Zebrafish	Impaired contractility;	-	
				Ventricular hypertrophy due to hyperplasia		
DNA Methylation	Dnmt1	Knockout	Mouse	Embryonic lethal by E11; Underdeveloped heart	-	1606615
	Dnmt3a	Knockout	Mouse cardiomyocytes and hESC	No effect on higher order chromatin	-	29162810
	Dnmt3b	Knockout	Mouse cardiomyocytes and hESC		-	
		Knockout	Mouse	Embryonic lethal by E16.5 Impaired septum formation; Hemorrhaging	-	16501171
		Targeted point mutations	Mouse	Normal development, small body size Thickening of myocardium in some animals	-	
		Cardiac specific knockout	Mouse	No effects in basal conditions. Blunted hypertrophic response after TAC or ISO treatment	Myh7 is alternatively spliced after Dnmt3 depletion	25784084
	MeCP2	Cardiac-specific overexpression	Mouse	Embryonic lethal by E15 Ventricular wall and septal hypertrophy due to hyperplasia	Changes in cardiac genes (e.g. increases in ANF, Mlc2v, Nkx2-5; decreases in βMHC, α-cardiac actin, Tbx5)	20203171
Chromatin Structural Proteins	Ctcf	Cardiac specific knockout	Mouse (Adult)	Hypertrophy and heart failure	Alteration of TAD boundary strength. Chromatin loop disruption. Decrease of the local intra-chromatin interactions. Alteration of enhancer-promoter interactions.	28802249
		Cardiac specific knockout	Mouse (Embryo)	Embryonic lethality after E12.5	Genes involved in mitochondrial function and the IrxA cluster.	28846746
		Knockdown	mESC	Cell death after 4 days of depletion	Loss of TAD Insulation.	28525758
		Gain and loss of function	C2C12	CTCF induces cell myogenic differentiation	Myogenic development genes (Myf5, MyoD and Myogenin) and Wnt signaling.	21288905
		Gain and loss o function	Zebrafish	CTCF induces myogenesis *in vivo*		
	Hmga2	Overexpression	P19CL6 cells	Efficient differentiation into cardiomyocytes	Upregulation of cardiac markers (Nkx2-5, ANP, Gata4, Mef2c, Mlc2v)	18425117
		Knockdown		Blocked cardiomyocyte differentiation	Downregulation of cardiac markers; Mesodermal markers not affected	
		Dominant negative mutation	Xenopus	Reduced heart size	Downregulation of Nkx2-5	
		Knockdown		--	Downregulation of Nkx2-5	
	Lamin A/C	Knockout	Mouse	Reduced growth rate (postnatal 2wks); Dilated cardiomyopathy and impaired ventricular contractile function (postnatal 4-6wks)	Upregulation of ANP and BNP; Downregulation of SERCA2a; Elongated nuclear shape, disrupted chromatin structure	14755333
	Nucleolin	Knockdown	Zebrafish	Edema; Small ventricular size; Impaired looping Decrease in cardiac output	Upregulation of p53; Decreased rRNA expression; Ventricular bmp4 not repressed; Increase in H3K9me3	24077883
		Overexpression		Edema; Impaired looping, dorsal-ventral axis formation	Upregulation of p53, ventricular Bmp4	
Lymphocyte Antigen	Ly75	Knockdown	Zebrafish	Heart failure with dilation of the atrium and reduced ventricular contractility.	-	23341106
(G protein)-coupled receptor	Adora2a	Knockdown	Zebrafish	Severe heart failure with progressively decreasing ventricular contractility	-	23341106
BET inhibitors	BET inhibitors	JQ1 inhibition	Rat and Mouse Cardiomyocytes	Left ventricular hypertrophy blocking	Calcineurin, NFκB, and GATA4	23939492, 23911322
		JQ1 inhibition	iPSC-CMs	Agonist-induced hypertrophy blocking	NFκB and TGF-β signaling networks	28515341

Supplemental Table I. Gain/Loss of Function Studies on Chromatin Modifiers in the Cardiovascular System.

LncRNA	Manipulation	Model	Phenotype	Molecular targets	Reference (PMID)
Braveheart	Knockout	mESC	Necessary for cardiovascular lineage commitment of embryonic stem cells	MesP1 and PRC2	23352431
Chaer	Knockout	Mouse	No noticeable phenotype Cardiac hypertrophy in response to pressure overload was significantly attenuated in the KO	PRC2	27618650
Chast	Loss and gain of function	Mouse	Overexpression induces hypertrophy, increased cardiomyocyte size and fibrosis. Silencing prevents and attenuates TAC-induced pathological cardiac remodeling	Chast activates Bnp, β-Mhc and Ctgf Chast negatively regulated Pleckstrin homology domain-containing protein family M member 1	26888430
Fendrr	Knockout	Mouse	Omphalocele and blood accumulation in the right heart chamber. Embryonic lethal around E13.75	PRC2, EZH2 and SUZ12	23369715
Hand2	Knockout	Mouse	Right ventricle hypoplasia and embryonic lethality	RNA polymerase II elongation	27783597
Mantis	Knockdown	HDLEC	Regulates endothelial angiogenesis	Brg1, SOX18, SMAD6, and COUP-TFII	28351900
Malat-1	Knockout	Mouse	No effect in cardiac hypertrophy upon pressure overload	mRNA splicing	26919721
Mhrt779	Overexpression	Mouse	Heart protection from hypertrophy and failure	It prevents Brg1 from recognizing its genomic DNA targets	25119045
Uph	Knockdown	Mouse	Right ventricular hypoplasia and embryonic lethality	Hand2	27783597

Supplemental Table I. Gain/Loss of Function Studies on Chromatin Modifiers in the Cardiovascular System.

Aco2	Mertk	Acss1	Pxk
Adcy6	Meis2	Kptn	Man1c1
Arvcf	Ndufs4	Med26	Arhgap35
Bad	Nxn	Sik3	Klc3
Bcat2	Serpinf2	Pitpnc1	Pla2g3
Bcl2	Ptp4a3	Dpep3	Fscn2
Bmpr2	Ptpn14	Nkx6-3	Setbp1
Bsn	Pxn	St5	Kcng2
Cct6a	Rai1	Klhl2	Pik3c2b
Cdh16	Rara	Ankrd17	D430041D05Rik
Ift81	Rgl2	Arid1a	2900026A02Rik
Celsr1	Nptn	Cnnm2	Map7d1
Inadl	Tgfbr3	Deptor	Mpg
Cnih2	Tns1	Phlpp1	Gm20735
Col13a1	Cdh23	Ehd4	Ablim3
Col14a1	Vav2	Abtb2	March3
Dapk2	Fmnl3	Slco2b1	Mical2
Des	Car14	Itga9	Lgr6
Dnase1l3	Pappa2	Bre	Zc3h4
Ebf1	Limd1	Ston2	Btbd16
Ehd1	Ldlrad4	Znrf1	Rnf150
Eif4ebp1	Nudt3	Socs7	Lrch1
Gab1	Bcam	Rassf3	6330403A02Rik
Gcgr	Pnkp	Zbtb7c	C730036E19Rik
Gnb1	Slc22a17	Pde2a	Itprip
Grk5	Fam129a	Daam1	Ly6g6f
Grb2	Osbpl1a	Rhot2	Trabd2b
Hhex	Dpysl5	Slc43a2	Gm15441
Itgb5	Nicn1	Stxbp6	Mir1946b
Kcnab3	Map1lc3b	Susd6	Mir1198
Meis1	Wipf2	Mfsd7c	Cbarp

Supplemental Table II. List of genes with detected significant (*q* < 0.01 Fit-Hi-C interactions between transcription start and end sites (shown in Figure 3).
