# Supplementary material for: Epigenomes in Cardiovascular Disease
Source: Circ Res. 2018 May 24;122(11):1586–607. doi: 10.1161/CIRCRESAHA.118.311597 (PMC5976233; doi:10.1161/CIRCRESAHA.118.311597)
Supplement: Supplementary file 2 [file res-122-1586-s002.pdf]

Glossary of Terms

histones: basic (in the chemical sense of the term) proteins that bind eukaryotic DNA as part of octomeric protein complexes called **nucleosomes**; **histone post-translational modification** (a.k.a. histone PTM, a.k.a. histone marks): covalent modifications, often to the unstructured tails of histone proteins that orient outside the nucleosome core and are thus accessible to enzymes (recent evidence suggests that structured cores of histones are also host to PTM); **DNA methylation**: modification to DNA backbone; in mammals, this usually occurs on cytosines that are followed by guanines, so-called **CpGs**; **non-coding RNA**: an RNA molecule that does not have a known functional role as mRNA or any of the multiple other functions of RNA in eukaryotes ("noncoding" is a misnomer and a remnant of the need to distinguish RNAs that were large by did not code polypeptides from traditional protein-coding mRNAs; noncoding RNAs exhibit secondary structure, have a wide range of functions and are no less coding than mRNA, tRNA, rRNA or so forth…they just lack large open reading frames [**ORF**; region of genome with codons conducive to polypeptide generation]; long noncoding RNAs (**lncRNA**) are >200 amino acids, do not [usually] contain ORFs and have been ascribed a wide range of functions in chromatin regulation and non-nuclear processes); **topologically associating domain** (**TAD**): region of chromatin with high level of connectedness, demarcated by two TAD boundaries (which are in turn defined by local rate of change in insulation score); **chromosome territory**: a phenomenon observed mainly by microscopy, in which interphase chromosomes tend to segregate apart from each other, favoring intra-chromosomal interactions over the inter-chromosomal kind; **chromatin writers, erasers and readers**: the enzymes (often multi-protein complexes) that deposit, remove and interpret histone PTMs, respectively; **enhancer**: a region of DNA that is sometimes transcribed and which positively influences the transcription of a nearby gene, usually involving the binding transcriptional co-activators, RNA pol II and/or histone PTMs associated with active transcription; **heterochromatin**: broadly construed, a region of the genome that is densely packaged and not available for transcription. Heterochromatin can be **facultative**, which describes a kind that may switch to a transcriptionally more active state under the right circumstances, and **constitutive**, which describes a kind that is thought to be virtually always silent; **euchromatin**: a region of the genome receptive to transcription; **transcription factor**: a protein or protein complex that binds DNA and facilitates transcription; **chromatin conformation capture**: a basket of techniques including Hi-C (unbiased analysis of entire genomes) and 3C/4C/5C (all of which examine selected region[s] of interest) used to examine endogenous chromatin architecture. DNA-DNA interactions are fixed and then determined using DNA sequencing and bioinformatics. The various techniques have strengths and weaknesses in terms of sensitivity, resolution, comprehensiveness and cost; **chromatin immunoprecipitation and DNA sequencing** (**ChIP-seq**): immunoprecipitation of a protein followed by DNA sequencing to determine where that protein binds the genome. There are many variations on this technique which can in principle examine any protein, although commonly examined proteins are transcription factors, histone PTMs and RNA polymerase; **bisulfite sequencing**: a method in which bisulfite treatment of DNA (which converts unmethylated cytosines to uracil, leaving methylated ones unaffected) combined with DNA sequencing can be used to determine the entire **DNA methylome** (defined as all methylated cytosines), in so-called whole genome bisulfite sequencing, or in a more rapid and cheaper iteration called reduced representational bisulfite sequencing, can reveal the methylation status of the majority of CpGs in and around genes in **CpG islands** (defined as regions of increased frequency of cytosine-guanine dinucleotides which appear in mammalian genomes around the TSS of genes); **transcriptional start site** (**TSS**): the site where transcription of a gene begins. Compare with **transcriptional end sites (TES)**; **ATP-dependent chromatin remodeler**: enzyme or protein complex that consumes ATP to reorganize nucleosomes, often preceding transcription; **histone deacetylase** (**HDAC**; a.k.a. lysine deacetylase, of which a subclass includes Sirtuins): a family of enzymes that remove acetyl groups from lysines (often histones); **histone acetyl transferase** (**HAT**; a.k.a. lysine acetyltransferase): a family of enzymes that add acetyl groups to lysines in proteins, often histones; **histone methyltransferase** (**HMT**): a family of enzymes that add methyl groups to lysines; **histone demethylases**: a family of enzymes that remove methyl groups from lysines; **chromatin isolation by**

RNA purification followed by DNA sequencing (**ChiRP-seq**): a technique wherein labeled (often biotinylated) RNA complementary to an RNA of interest is used, along with DNA sequencing, to determine regions of the genome bound by the target RNA; **imprinting**: allele-specific gene expression controlled by non-genetic factors such as DNA methylation and maybe histone modifications; **Trithorax group/MLL protein complexes (TrxG/MLL)**: a class of well-studied complexes responsible for activating histone marks; **Polycomb repressive complex (PRC)**: a class of well-studied complexes responsible for repressive histone marks; formaldehyde assisted interrogation of regulatory elements (**FAIRE**), **DNAse I** digestion for the production of "hypersensitivity sites", assay for transposase accessible chromatin (**ATAC**), and micrococcal nuclease digestion (**MNase**) are a series of techniques that distinguish open/active chromatin from restricted/inactive chromatin and, when combined with DNA sequencing, do so in a genome-wide manner; BRG1: Brahma-related gene 1, the ATPase subunit of the BAF ATP-dependent chromatin remodeling complex in mammals; SWItch/Sucrose Non-Fermentable (**SWI/SNF**): an ATP-dependent chromatin remodeling complex in yeast, of which BRG1 is a functional homolog; BAF; Brahma associate factor, an ATP-dependent chromatin remodeling complex in mammals, consisting of >10 subunits; **ChIP-exo**: ChIP combined with exonuclease treatment to generate smaller fragments for subsequent sequencing, increasing resolution in subsequent informatics analysis of protein binding; **bivalency**: the presence of active and repressive marks on the same region of chromatin, thought to be associated with genes poised for activation; **DamID**: DNA adenine methyltransferase identification, a technique using the bacterial Dam protein fused to a protein of interest to determine where the latter binds the genome by virtue of its conjugation to the former, which methylates adenine as a diagnostic signature; **FISH**: fluorescence *in situ* hybridization, a technique enabling microscopic detection of specific DNA or RNA molecules using fluorescent tagged nucleotide probes complementary to the sequences of interest; **epigenome**: a genome plus everything bound to it.
